# Nitrogen fertilization and rhizosphere processes regulate methane uptake in a nitrogen-limited forest

**DOI:** 10.3389/fpls.2026.1719243

**Published:** 2026-04-20

**Authors:** Jianyu Chen, Ying Deng, Chao Wang

**Affiliations:** 1 School of Life Science and Technology, Northwestern Polytechnical University, Xi’an, China; 2 State Key Laboratory of Vegetation and Environmental Change, Institute of Botany, Chinese Academy of Sciences, Beijing, China

**Keywords:** larch plantation, meta-analysis, methane uptake, nitrogen addition, rhizosphere, temperate forest

## Abstract

Methane (CH_4_), uptake by well-drained soils plays a vital role in mitigating atmospheric CH_4_. Previous research has demonstrated that nitrogen (N) addition and rhizosphere processes markedly affect soil CH_4_ uptake. However, how rhizosphere activity mediates the response of soil CH_4_ uptake to N addition remains unclear. Here, we conducted *in-situ* measurements of CH_4_ fluxes using collar treatments to quantitatively assess the rhizosphere contribution to soil CH_4_ uptake in a temperate larch plantation. We further evaluated the effects of N addition level (CK: no N addition; low-level N addition: 20 kg N ha^-1^ yr^-1^; high-level N addition: 50 kg N ha^-1^ yr^-1^) and duration (short-term and long-term) on CH_4_ uptake. We found that soil CH_4_ uptake was insensitive to low-level N addition but significantly reduced by 20.9% under high-level N addition. Notably, rhizosphere CH_4_ uptake decreased even under low-level N addition, primarily due to reduced rhizosphere activity in our N-limited plantation. A global meta-analysis further confirmed that N addition below 25 kg N ha^-1^ yr^-1^ had no effect on soil CH_4_, whereas higher N levels suppressed it by increasing soil NH_4_^+^-N and NO_3_^-^-N accumulation. Both field experiments and meta-analysis found no significant effect of N addition duration on soil CH_4_ uptake. Overall, our study highlights the critical role of rhizosphere processes in regulating the soil CH_4_ uptake under N addition highlight differential responses to N addition levels, and provide important implications for predicting forest soil CH_4_ sinks under future global N deposition scenarios.

## Introduction

Methane (CH_4_) is a potent greenhouse gas that contributes approximately 20% of the total radiative forcing from long-lived greenhouse gases, with a global warming potential 28 times higher than that of carbon dioxide (CO_2_) over a 100-year timeframe (Etminan et al., 2016; Saunois et al., 2020). Forest soils act as major terrestrial CH_4_ sinks, oxidizing approximately 33.5 ± 0.6 Tg CH_4_ yr^-1^ globally, with well-aerated soils exhibiting the greatest uptake rates (Aronson et al., 2013; Murguia-Flores et al., 2018). However, the response of the global CH_4_ sink strength in forest soils to environmental changes remains highly uncertain, which may be attributed to limited understanding of soil nitrogen (N) availability and rhizosphere activity (Li et al., 2022; Maier et al., 2017; Tamale et al., 2021). Increasing atmospheric N deposition, resulting from anthropogenic activities, can modify soil CH_4_ uptake by altering methanotrophic activity and rhizosphere carbon dynamics (Ackerman et al., 2019; Sullivan et al., 2013; Fransson et al., 2016). Elucidating the mechanisms by which N availability regulates CH_4_ uptake is critical for refining terrestrial CH_4_ budgets and informing forest management strategies.

Literature on the effect of N addition on soil CH_4_ uptake are inconsistent, with studies showing inhibition, no effect, or stimulation depending on N levels, duration, and soil conditions (Aronson and Helliker, 2010; Gong et al., 2021; Chen et al., 2023; Gao et al., 2023; Liu and Greaver, 2009). At low levels, N addition can alleviate N limitation in methanotrophic communities, thereby enhancing CH_4_ uptake in N-limited soils (Bodelier and Laanbroek, 2004), (Xu et al., 2014). In contrast, high N inputs can suppress CH_4_ uptake through soil acidification, competitive inhibition of methane monooxygenase by NH_4_^+^, or toxicity from nitrite (NO_2_^-^) and hydroxylamine (NH_2_OH) (Nyerges and Stein, 2009). Moreover, long-term N addition may further shift methanotroph communities from high-affinity type II to low-affinity type I methanotrophs (Chan et al., 2005). These discrepancies likely reflect differences in soil N status, methanotrophic community structure and climatic conditions, underscoring the need for mechanistic field studies across diverse forest ecosystems.

Methanotrophs, the primary drivers of soil CH_4_ uptake, are facultative bacteria capable of utilizing low molecular weight compounds (i.e. methanol) exuded by roots and mycorrhizal hyphae (Bodelier and Laanbroek, 2004; Praeg et al., 2016). Rhizosphere processes can account for up to 64% of soil CH_4_ uptake in temperate forests by supplying labile C that stimulates methanotrophic activity under low CH_4_ availability (Subke et al., 2018). However, N enrichment may alter rhizosphere exudates and mycorrhizal associations, thereby indirectly suppressing CH_4_ uptake through changes in methanotrophic abundance and activity. For example, N-induced changes in plant communities can reduce *pmoA* gene abundance via rhizosphere pathways (Chen et al., 2023). Carbohydrates are preferentially allocated to the rhizosphere to support N assimilation under N-limited soils (Du and Fang, 2014; Hogberg et al., 2001). When soil N availability increases, plants tend to invest fewer photosynthates into root systems, this reduction in the photosynthates can, in turn, suppress CH_4_ uptake (Yan et al., 2018). However, the rhizosphere-mediated response to N enrichment is likely more complex, involving both this indirect C-limitation pathway and the direct inhibition of methanotrophic activity by N availability. Yet, the magnitude and mechanisms of rhizosphere-mediated CH_4_ uptake responses to N addition remain insufficiently understood, particularly in temperate plantation forests (Subke et al., 2018).

Over the past three decades, plantation forests have expanded rapidly, particularly across temperate regions such as China (Payn et al., 2015). Larch (Larix spp.), a key conifer species, is widely distributed throughout temperate and boreal zones (Yan et al., 2019). In Northeast China, for instance, larch plantations now cover approximately 2.61 × 10^6^ ha (Chinese Ministry of Forestry, 2014). In this study, we conducted a three-year (2017-2019) field experiment in a larch (*Larix principis-rupprechtii*) plantation in Saihanba, northern China and combined with a global meta-analysis. Given the strong dependence of methanotrophs on root-derived carbon and their sensitivity to N addition, our objectives were to (1) quantify the contribution of rhizosphere process to soil CH_4_ uptake; (2) assess how N addition level and duration influence soil CH_4_ uptake; and (3) test whether N enrichment suppresses rhizosphere CH_4_ uptake directly or indirectly via altered plant carbon allocation. By integrating site-specific measurements with global patterns, this study aims to advance mechanistic understanding of CH_4_ dynamics and inform management strategies for enhancing CH_4_ sinks in temperate plantation forests.

## Materials and methods

### Field experiment

The study was conducted in a 50-year-old larch (*Larix principis-rupprechtii*) plantation at the Saihanba Ecological Station of Peking University (42°04’-42°36’ N, 116°53’-117°38’ E, 1505 m a.s.l), located in Saihanba National Forest Park, Hebei Province, China (http://www.saihanba.pku.edu.cn/). The region transitional between temperate deciduous broad-leaved forest and temperate grassland, and the experimental site is located in the semi-humid region. The mean annual temperature is -1.4 °C (1971-2010). The mean annual precipitation is 450 mm, with 68% occurring from June to August (Ma et al., 2014). Snowfall typically begins in October, with snow depth less than 30 cm in winter. Ambient N deposition is approximately 13 kg N ha^-1^ yr^-1^ (Sun et al., 2016). Soil is well-drained and sandy (54-74% sand), and the topography is flat. The stands were thinned in 1989.

Historically, this area experienced large-scale industrial logging during the 20th century, after which it was replaced by secondary forests and plantations (Wang et al., 2010). The Saihanba plantation, established in 1962, now covers 94700 ha and is the largest plantation in China with dominant species of *L. principis-rupprechtii* and *Pinus sylvestris* var. *mongolica*.

### Experimental design

#### Long-term N addition experimental

In August 2009, site A (100 × 100 m) was fenced to minimize disturbance (**Supplementary Figure 1**). In May 2010, nine 20 × 20 m plots were established, with three replicates for each N addition treatment: control (CK, no N addition), low-level N addition (N20, 20 kg N ha^-1^ yr^-1^), and high-level N addition (N50, 50 kg N ha^-1^ yr^-1^). Plots were separated by >10 m buffer strips. Nitrogen was applied as urea solution via backpack sprayers, with six applications annually (monthly from May to October). Each application added water equivalent to 0.0625 mm rainfall, and the same amount of water was added to the CK plots.

To assess rhizosphere contributions to CH_4_ uptake and soil respiration, we established three treatments with different heights of polyvinyl chloride (PVC) collars (Sun et al., 2014; Yan et al., 2018).

To measure both soil CH_4_ uptake (SR treatment, including roots and bulk soil) and soil respiration. The first type of PVC collars (20 cm diameter and 11 cm height) was inserted 8 cm into the soil and installed at randomly selected positions in each plot. The depth of the litter layer could reach 5 cm, only roots at the top 3 cm were potentially affected, assumed to have negligible impact on CH_4_ and CO_2_ fluxes (Shah et al., 2025).

To measure both bulk CH_4_ uptake (S treatment, bulk soil only) and heterotrophic respiration, we installed second type of PVC collars (20 cm diameter, 50 cm height) in each plot. Collars were inserted into the soil to a depth of 47 cm to cut the roots in each site and that excluded roots, as prior studies confirmed minimal roots below 35 cm in sandy soils (Sun et al., 2014).

Both rhizosphere CH_4_ uptake (R treatment, root-only) and rhizosphere respiration was calculated as the difference between SR and S treatments.

In total, 54 collars (9 plots × 3 collar groups per plot × 2 collars [SR and S] per group) were randomly installed, with collars spaced ≥ 2 m apart. All collars were permanently left in place throughout the measurement period, and any living plants inside collars were manually removed each week.

#### Short-term N addition experimental

To evaluate the effect of N addition duration on soil CH_4_ uptake (SR treatment), site B was established in May 2018, approximately 20 m from site A (**Supplementary Figure 1**). Prior to the experiment, there were no significant differences in initial vegetation, soil properties, or soil CH_4_ uptake between site B and the control plots of site A (**Supplementary Table 1**). The experimental design and N addition method were identical to site A, except that the experiment at site B began in 2018.

At site B, only soil CH_4_ uptake (SR treatment, including roots and bulk soil) was measured. The first type of PVC collars (20 cm diameter, 11 cm height) was used, with a total of 18 collars (6 plots × 3 collars per plot) installed at randomly selected positions within site B.

### Soil CH⁠_4_ and CO_2_ fluxes measurements

Soil CH_4_ and CO_2_ fluxes were measured twice monthly during the growing season (May to October) using a Fast Greenhouse Gas Analyzer (FGGA, Model915-0011, Los Gatos Research, CA, USA) at site A (2017-2019) and site B (2018-2019). The gas chamber was placed tightly over PVC collars before measurements. During the measurements, the rate of change of CH_4_ and CO_2_ concentration within the gas chamber was calculated by linear regression, and then the CH_4_ and CO_2_ flux was calculated by the rate of CH_4_ and CO_2_ concentration change using the following equation (Stiles et al., 2018):

(1) $$F=S\frac{V\;M\;273.16}{A\;Vm(273.16+T)}60$$

Where:

F = Soil CH_4_ (nmol CH_4_ m^-2^ s^-1^) and CO_2_ (μmol CO_2_ m^-2^ s^-1^) flux

S = Rate of change in CH_4_ (ppb s^-1^) and CO_2_ concentration (ppm s^-1^)

V = Chamber volume (m^3^)

A = Chamber site (m^2^)

M = Molecular mass of CH_4_ and CO_2_ (g mol^-1^)

V_m_ = Ideal gas mole volume (0.0224 m^3^ mol^-1^)

T = Air temperature inside the chamber (°C)

With Equation 1, data from individual measurements were excluded if the regression coefficients (R^2^) were below 0.9 (Stiles et al., 2018). Soil temperature and volumetric moisture were measured at a depth of 5 cm near each PVC collar by using sensors integrated with the FGGA.

### Sampling and measurements

#### Soil samples

Soil samples were collected in early July and early September (peak and late growing season) during 2017–2019 at site A, and 2018–2019 at site B. Within each plot, soil was sampled at five locations to a depth of 0–30 cm using a 4 cm diameter auger. Samples were immediately transferred to plastic bags inside an icebox and transported to the laboratory. The five subsamples from each plot were combined to form a composite sample and passed through a 2 mm sieve to remove plant residues, roots, gravels and stones. Each soil sample was separated into four subsamples for different analyses. The first sample was air dried and transported to the laboratory to measure the soil pH; the second sample was used to determine the soil organic carbon (SOC) and soil total nitrogen (TN); the third sample was used to determine the NH_4_^+^-N and NO_3_^-^-N concentrations within 24 h; and the fourth sample was stored at 4 °C for determinations of the dissolved organic carbon (DOC), microbial biomass carbon (MBC) and microbial biomass N (MBN) levels.

Soil pH was measured in a 1:5 soil: deionized water suspension using a pH Meter (PHSJ-4A, INESA, Shanghai, China). To measure the SOC and TN, the soil samples (15 g each) were measured by a total organic carbon analyzer (TOC-CPN, Shimadzu, Japan). Soil samples were extracted with 50 mL 2 M KCl in a 50 mL plastic bottle on a rotary shaker (200 r/min) for 1 h, then the extracts were filtered and analyzed for NH_4_^+^ and NO_3_^-^ using a CNS elemental analyzer (Variomax CNS Analyser, Elementar GmbH, Hanau, Germany). The DOC was extracted by 0.05 mol L^-1^ K_2_SO_4_ solution then using a total organic carbon analyzer (TOC-CPN, Shimadzu, Japan). MBC and MBN were determined using the chloroform fumigation extraction method (Vance et al., 1987). MBC and MBN concentrations were corrected using correction factors of 0.45 for C and 0.54 for N (Brookes et al., 1985).

Rhizosphere soil was collected in early September during 2017–2019 at site A. A 20 × 20 × 20 cm volume of soil containing the root system was collected per plot. Soil adhering to fine roots after gentle shaking was considered rhizosphere soil (Sun et al., 2021) and collected carefully with soft brushes for DOC analysis.

#### Plant samples

Root samples were collected at the same time points as soil samples at site A. Five soil cores (0–30 cm depth, 4 cm diameter) were collected per plot. Roots were then divided into fine roots (< 2 mm in diameter) and coarse roots (> 2 mm), oven dried at 65 °C for 48 h, and weighed to calculate the root biomass. Carbon and nitrogen concentrations were measured using an elemental analyzer (2400 II CHNS/O, Perkin-Elmer, USA).

In site A, two 1 × 1 m litter traps (0.5 mm mesh, 0.8 m height) were randomly placed per plot. The traps were emptied every two months during the growing season. Litterfall was oven dried at 65 °C for 48 h and then weighed. Annual litterfall mass was estimated as the mean of the two traps per plot.

### Statistical analysis

Data normality was assessed using the Kolmogorov-Smirnov test (*P* > 0.05). One-way analysis of variance (ANOVA) was used to examine the effects of N addition on CH_4_ uptake (SR, R and S treatment), soil temperature, soil moisture, soil properties, soil respiration, root properties and litterfall. Linear mixed effect models (LMMs) were applied to test the effects of N addition, year and their interactions on soil CH_4_ uptake (SR, R and S treatment), with N addition and year treated as fixed effects and plot as a random effect. Linear regression analyses were conducted to quantify the relationships between CH_4_ uptake (SR, R and S treatment) and soil properties, environmental variables, soil respiration, root properties and litterfall. Random Forest models were applied to identify key predictors of soil CH_4_ uptake (SR, R and S treatment), with variable importance assessed by the percentage increase in mean squared error (%IncMSE) using the ‘randomForest’ package in R v4.2.2. Predictor significance was tested via permutation using the ‘rfPermute’ package (Cui et al., 2025). Structural equation models (SEM) were applied to elucidate the direct and indirect biotic and abiotic variables of soil CH_4_ uptake (SR and R treatment). All statistical analyses were conducted using R v4.2.2. Differences were considered statistically significant at *P*< 0.05. Values are presented as mean ± standard error unless otherwise specified.

### Meta-analysis

#### Database compilation

In addition to our field experiment, we conducted a literature review of peer-reviewed articles published up to September 2025 to assess the effects of N addition duration on temperate forest CH_4_ fluxes. All published data were derived from Web of Science (WoS), Google Scholar, and the China Knowledge Resource Integrated Database (CKRI). All the specified languages in the search are English. Search terms included “CH_4_” OR “methane” OR “GHG” OR “greenhouse gas” AND “N” OR “nitrogen” OR “NH_4_” OR “NH_3_” OR “ammonium” OR “nitrate” AND “addition” OR “deposition” OR “application”. Studies were screened using five criteria: (1) Both control and treatment experiments were included; (2) N addition experiments were conducted in the field rather than in laboratory incubations; (3) N addition duration should be longer than a growing season or a full year; (4) The data obtained under different N addition durations, levels, forms, and experimental locations were independent; (5) Multiple experiments may be available in a single paper, but only the data from the N addition experiment (i.e., treatment group) and its control experiment were extracted. Based on these criteria, we compiled a global dataset of 57 soil CH_4_ fluxes observations from 21 studies (**Supplementary Table 2**).

We extracted all necessary data from the textual descriptions, tables, and graphical representations. Where data were presented solely in graphical form, we digitized the data points using OriginPro 2017C software (https://www.OriginLab.com). All the collected data were converted to common units. Notebly, we only collected data from the top soil layer (0–10 cm), as the majority of CH_4_ consumption by methanotrophs occurs near the soil surface.

To investigate the influence of experimental conditions, N addition duration was categorized into two groups based on previous studies and our field experiment (Lu et al., 2021; Wu et al., 2022): short-term (< 3 years) and long-term (≥ 3 years).

### Data analysis

The meta-analysis method proposed by Hedges et al. (1999) was adopted because it can quantitatively evaluate the response of CH_4_ uptake to N addition. Herein, the response of CH_4_ uptake to N addition is quantified by a natural log-transformed response ratio (*RR*), which is defined as the ratio of the mean of selected variable at the treatment group ( $\bar{X_{t}}$) to that at the control group ( $\bar{X_{c}}$):

(2) $$\ln RR=\ln\frac{\bar{X_{t}}}{\bar{X_{c}}}=\ln\bar{X_{t}}-\ln X_{c}$$

Where $\bar{X_{t}}$ and $\bar{X_{c}}$could be any variable to be compared. Negative values of *RR* are excluded because of the characteristics of above logarithm evaluation.

Based on this definition, the variance (v) of *RR* can be calculated as:

(3) $$v=\frac{s_{t}^{2}}{n_{t}{\bar{X_{t}}}^{2}}+\frac{s_{c}^{2}}{n_{c}{\bar{X_{c}}}^{2}}$$

where $s_{t}$ and $s_{c}$ are standard deviations of treatment and control groups, respectively; $n_{t}$ and $n_{c}$ are sample sizes of treatment and control groups, respectively. The reciprocal of this variance is defined as the weighting factor to average all individual observations, i.e.,

(4) $$w_{ij}=\frac{1}{v}$$

where *i* is the group index, and *j* is the observation index at the *i*-th group.

With Equations 2–4, the weighted response ratio (denoted as *RR*_++_), its standard error (*S*(*RR*_++_)), and its 95% confidence interval (CI) were calculated as follows:

(5) $$RR_{++}=\frac{\sum_{i=1}^{m}\sum_{j=1}^{k_{i}}w_{ij}\ln RR_{ij}}{\sum_{i=1}^{m}\sum_{j=1}^{k_{i}}w_{ij}}$$

(6) $$S(RR_{++})=\sqrt{\frac{1}{\sum_{i=1}^{m}\sum_{j=1}^{k_{i}}w_{ij}}}$$

(7) $$95\%CI=RR_{++}\pm 1.96S(RR_{++})$$

where *m* is the number of groups (classified according to ecosystem types, N additional levels, durations or forms), *ki* is the number of observations at the *i* th group. Equations 5–7 were computed by using MetaWin software 2.1 (Sinauer Associates, Inc. Sunderland, MA, USA).

The percentage change (%) transformed from the weighted response ratio (*RR*_++_) is used to quantify the magnitude of the impacts of N addition:

(8) $$percentage\;(\%)=[\exp(RR_{++})-1]\times 100\%$$

If the 95% *CI* of *RR*_++_ of a selected variable does not overlap zero, the impact of N addition on this variable is considered as significant at *P*< 0.05 (Equation 8). Furthermore, between-group heterogeneity tests (Q_B_ tests) are conducted to analyze whether there is a statistical difference among the impacts of N addition in different groups.

## Results

### Responses of biotic and abiotic factors to N addition

Soil temperature in the larch plantation peaked in June-August (24.2 ± 2 °C) and declined by early October (5.6 ± 1.8 °C) (**Supplementary Figure 2**). No significant differences were observed among years or N treatments (*P* > 0.05; **Table 1**). Soil moisture varied significantly across years (*P*< 0.05; **Supplementary Figure 2**), being lowest in 2019, but showed no N treatment effects (*P* > 0.05; **Table 1**; **Supplementary Figure 2**).

**Table 1:** Effects of N addition on biotic and abiotic properties at site A (9-year N addition) and site B (2-year N addition).

Variables	Site A (long-term N addition)			Site B (short-term N addition)	
	CK	N20	N50	N20	N50
Soil temperature (°C)	17.45 ± 0.20	17.66 ± 0.14	17.33 ± 0.16	17.05 ± 0.30	16.88 ± 0.27
Soil moisture (%)	5.77 ± 0.09	5.72 ± 0.08	5.78 ± 0.09	5.42 ± 0.09	5.58 ± 0.11
NH_4_^+^-N (mg L^-1^)	8.12 ± 0.42^a^	9.14 ± 0.51^abA^	12.20 ± 0.57^b^	9.62 ± 0.42^abB^	12.37 ± 0.77^b^
NO_3_^-^-N (mg L^-1^)	1.19 ± 0.15^a^	2.03 ± 0.2^bA^	2.99 ± 0.12^c^	2.63 ± 0.24^cB^	2.73 ± 0.42^c^
SOC (mg g^-1^)	14.84 ± 0.71	15.51 ± 0.95	16.55 ± 2.53	13.63 ± 0.97	14.66 ± 1.30
DOC (mg kg^-1^)	170.5 ± 7.7^ab^	178.6 ± 8.5^a^	165.1 ± 11.7^ab^	179.2 ± 11.4^a^	159.0 ± 6.9^b^
DOC_root_(mg kg^-1^)	216.9 ± 13.6^a^	196.1 ± 14.5^b^	187.7 ± 12.8^b^	/	/
TN (mg g^-1^)	0.93 ± 0.11	0.97 ± 0.13	1.05 ± 0.14	0.98 ± 0.15	1.17 ± 0.14
pH	6.51 ± 0.14^a^	6.30 ± 0.11^ab^	6.01 ± 0.13^b^	6.23 ± 0.10^ab^	5.97 ± 0.09^b^
MBC (mg L^-1^)	13.64 ± 1.88^a^	12.80 ± 1.82	11.15 ± 1.59	14.48 ± 1.41^b^	11.84 ± 1.49
MBN (mg L^-1^)	1.13 ± 0.15	1.21 ± 0.14	0.87 ± 0.09	1.30 ± 0.13	1.09 ± 0.17
MBC: MBN	11.71 ± 0.97 a	11.25 ± 0.91^a^	14.01 ± 1.36^b^	12.44 ± 1.15^ab^	12.14 ± 1.39^ab^
Soil respiration (umol m^-2^ s^-1^)	1.74 ± 0.07^a^	1.65 ± 0.08^ab^	1.33 ± 0.8^b^	1.54 ± 0.09^b^	1.48 ± 0.09^b^
Rhizosphere respiration (umol m^-2^ s^-1^)	0.37 ± 0.05^a^	0.35 ± 0.06^ab^	0.22 ± 0.05^b^	/	/
Heterotrophic respiration (umol m^-2^ s^-1^)	1.37 ± 0.06^a^	1.30 ± 0.06^ab^	1.11 ± 0.05^ab^	/	/
Fine root biomass (g m^-2^)	324.3 ± 11.7^a^	270.9 ± 10.6^b^	277.4 ± 13.9^b^	/	/
Coarse root biomass (g m^-2^)	88.30 ± 8.42	92.56 ± 9.08	94.73 ± 10.65	/	/
Total root biomass (g m^-2^)	412.6 ± 17.1^a^	363.4 ± 16.2^b^	372.1 ± 15.9^b^	/	/
Root carbon concentration (%)	30.55 ± 1.44^a^	32.51 ± 1.29^ab^	34.04 ± 1.52^b^	/	/
Root nitrogen concentration (%)	1.09 ± 0.05	1.15 ± 0.07	1.12 ± 0.08	/	/
Litter mass (g m^-2^ yr^-1^)	366.8 ± 25.1	370.9 ± 34.9	395.2 ± 27.7	/	/

Values are Means ± SE (n = 6). Different capital and lowercase letters denote significant differences between N addition duration and level (one-way ANOVA, *post hoc* LSD test, *P*< 0.05). SOC, soil organic carbon; DOC, dissolved organic carbon; DOC_root_: dissolved organic carbon in rhizosphere soil; TC, soil total carbon; TN, soil total nitrogen; NH_4_^+^-N, soil ammonium content; NO_3_^-^-N, soil nitrate content; MBC, microbial biomass C; MBN, microbial biomass N.

At site A (long-term N addition, 9-year), N addition significantly altered soil properties (**Table 1**). NH_4_^+^-N, NO_3_^-^-N, and SOC significantly increased by 50.2%, 151.3% and 25.1% under high-level N addition (N50), respectively (*P*< 0.05). Low-level N addition (N20) showed a stronger positive effect on NO_3_^-^-N and DOC (*P*< 0.05). In contrast, soil pH and DOC_root_ significantly decreased under N addition (*P*< 0.05). Regarding root properties, both fine root biomass and total root biomass declined by 9.8-16.4% under N addition (*P*< 0.05), whereas root carbon concentration significantly increased with increasing N addition level (*P*< 0.05). High-level N addition further significantly decreased soil respiration and rhizosphere respiration by 11.5% and 30.4%, respectively (*P*< 0.05).

At site B (short-term N addition, 2-year), N addition significantly increased NH_4_^+^-N and NO_3_^-^-N by 43.1%-129.4% (*P*< 0.05; **Table 1**), and enhanced MBC and SOC under N20 (*P*< 0.05). Soil pH decreased significantly under N50 (*P*< 0.05). Moreover, soil respiration showed significant decreases across N addition levels (*P*< 0.05).

### Effects of N addition on soil CH_4_ uptake

**Figure 1:**
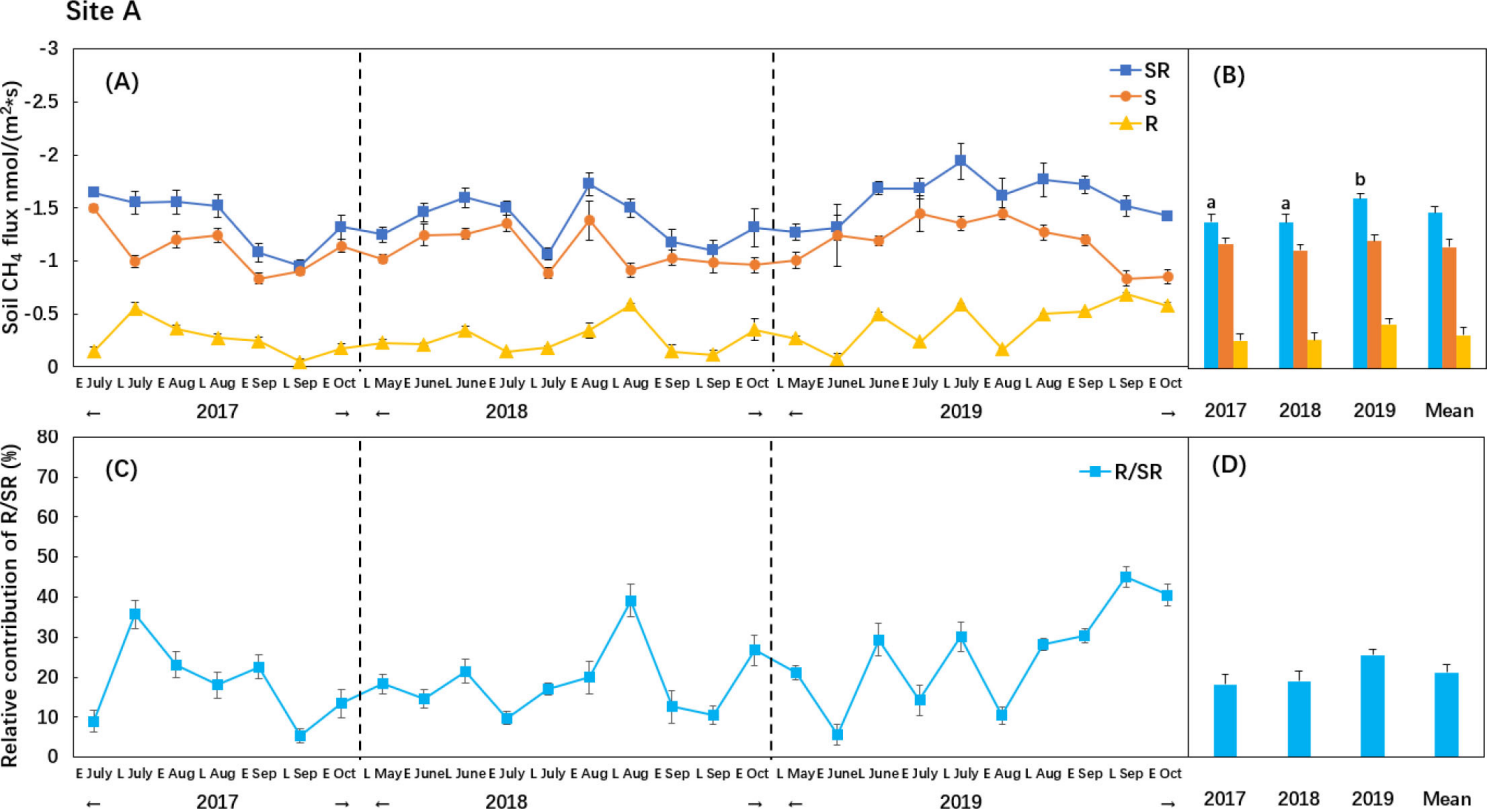
Temporal variation of CH_4_ uptake at the SR, R and S treatments in site A **(A)** and relative contribution of R treatment to SR treatment **(C)** and their annual means **(B, D)** at the site A. The error bars indicate the standard error of means. Different lowercase letters denote significant differences between years (one-way ANOVA, post hoc LSD test, *P* < 0.05).

**Figure 2:**
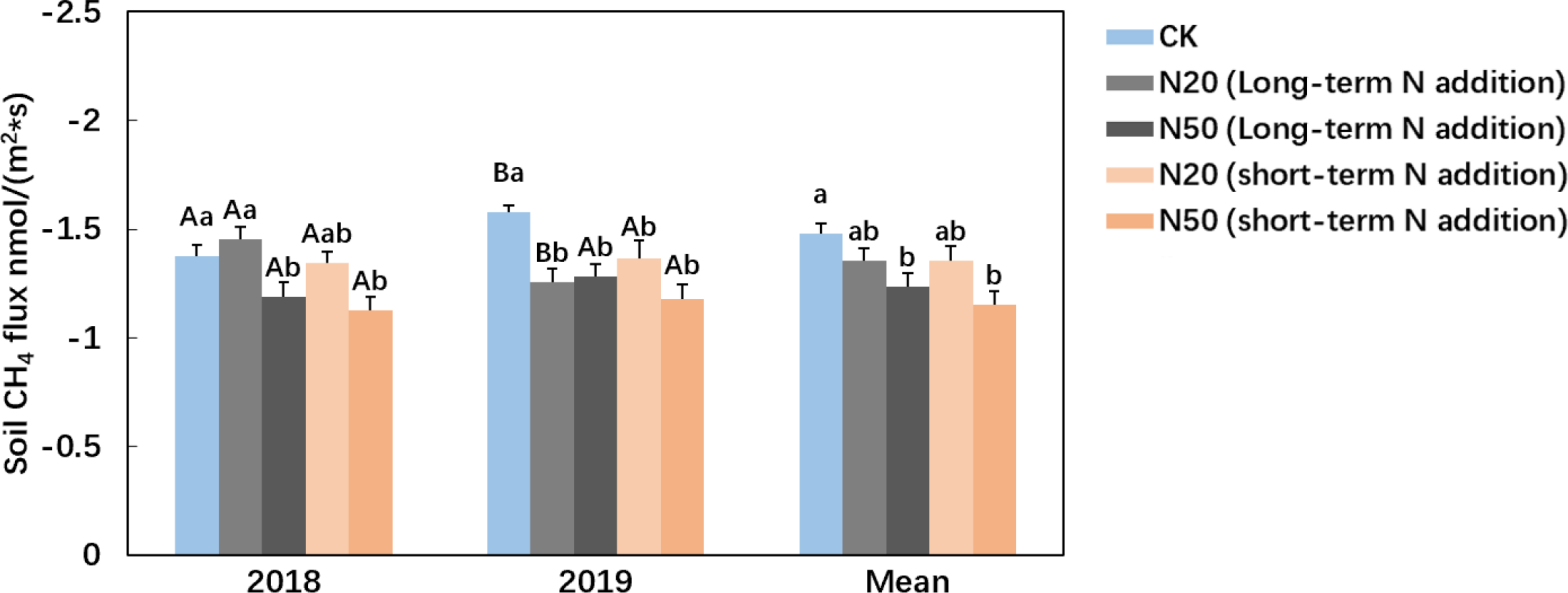
Effects of N addition duration (Long-term N addition, site A & Short-term N addition, site B) on soil CH_4_ uptake (SR treatment) in larch plantation. CK, N20 and N50 stand for ambient 0, 20 and 50 kg N ha^-1^ yr^-1^ N addition, respectively. The error bars indicate the standard error of means. Different lowercase letters denote significant difference between N addition level. Different upcase letters denote significant difference between years (one-way ANOVA, *post hoc* LSD test, *P*< 0.05).

At site A, soil CH_4_ uptake (SR treatment, including roots and bulk soil) averaged 1.49 ± 0.07 nmol m^-2^ s^-1^, peaking in summer and declining in the early and late growing seasons (**Figure 1**). Low-level N addition showed no significant effect on soil CH_4_ uptake (SR treatment), whereas high-level N addition significantly decreased soil CH_4_ uptake by 20.8% (**Table 2**; **Supplementary Figure 3**). The response of bulk CH_4_ uptake (S treatment) to N addition was similar with soil CH_4_ uptake (SR treatment) (**Table 2**). At site B, N20 and N50 reduced soil CH_4_ uptake (SR treatment) by 8.3% and 21.9%, respectively. The effect of N addition on soil CH_4_ uptake showed no significant difference between site A and site B (**Figure 2**).

**Table 2:** Effects of N addition on CH_4_ fluxes under (a) SR treatment, (b) S treatment, (c) R treatment and (d) relative contribution of R treatment to SR treatment at site A during 2017-2019.

Treatment	Mean CH_4_ flux (nmol m^-2^ s^-1^) at site A		
	CK	N20	N50
SR	-1.49 ± 0.07^a^	-1.32 ± 0.08^ab^	-1.18 ± 0.06^b^
R	-0.34 ± 0.04^a^	-0.21 ± 0.04^b^	-0.23 ± 0.04^b^
S	-1.15 ± 0.06^a^	-1.11 ± 0.07^ab^	-0.95 ± 0.08^b^
R/SR	22.73 ± 2.83% ^a^	15.26 ± 3.27% ^b^	20.27 ± 3.13% ^a^

CK, N20 and N50 stand for ambient 0, 20 and 50 kg N ha^-1^ yr^-1^ N addition, respectively. Different lowercase letters denote significant difference between treatments (one-way ANOVA, *post hoc* LSD test, *P*< 0.05). Values are Means ± SE.

**Figure 3:**
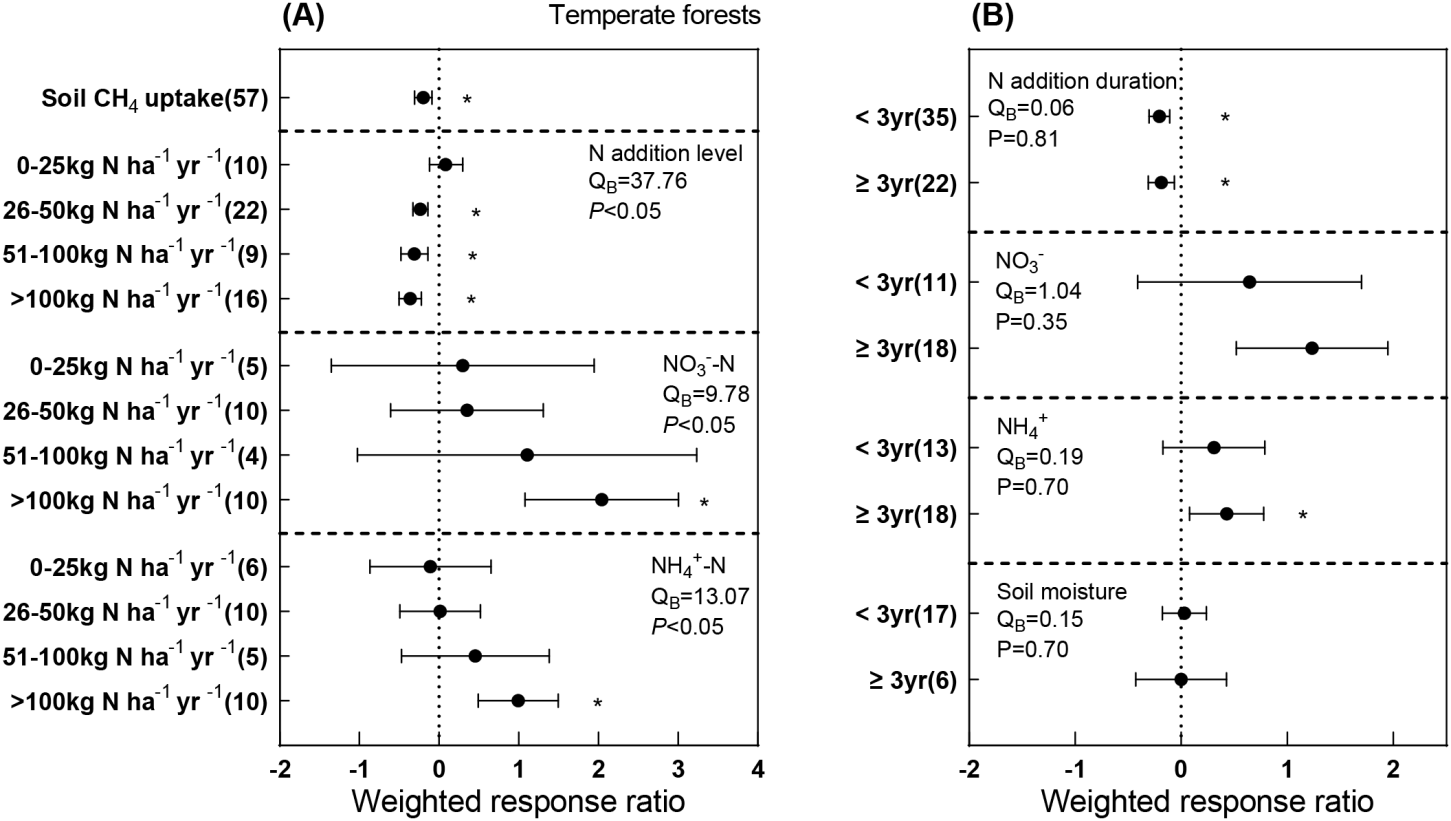
Meta-analysis of the effects of N addition on soil CH_4_ uptake and soil properties across different N addition level **(A)** and duration **(B)** in temperate forest. Left and right whiskers represent 95% CIs of weighted RRs, If 95% CIs did not overlap zero, the N addition effect was considered significant (*P*< 0.05, denoted by ∗). The numbers in parentheses represent the sample sizes of observations. Q_B_ indicates the level of differences of RRs among different N addition level and N addition duration.

**Figure 4:**
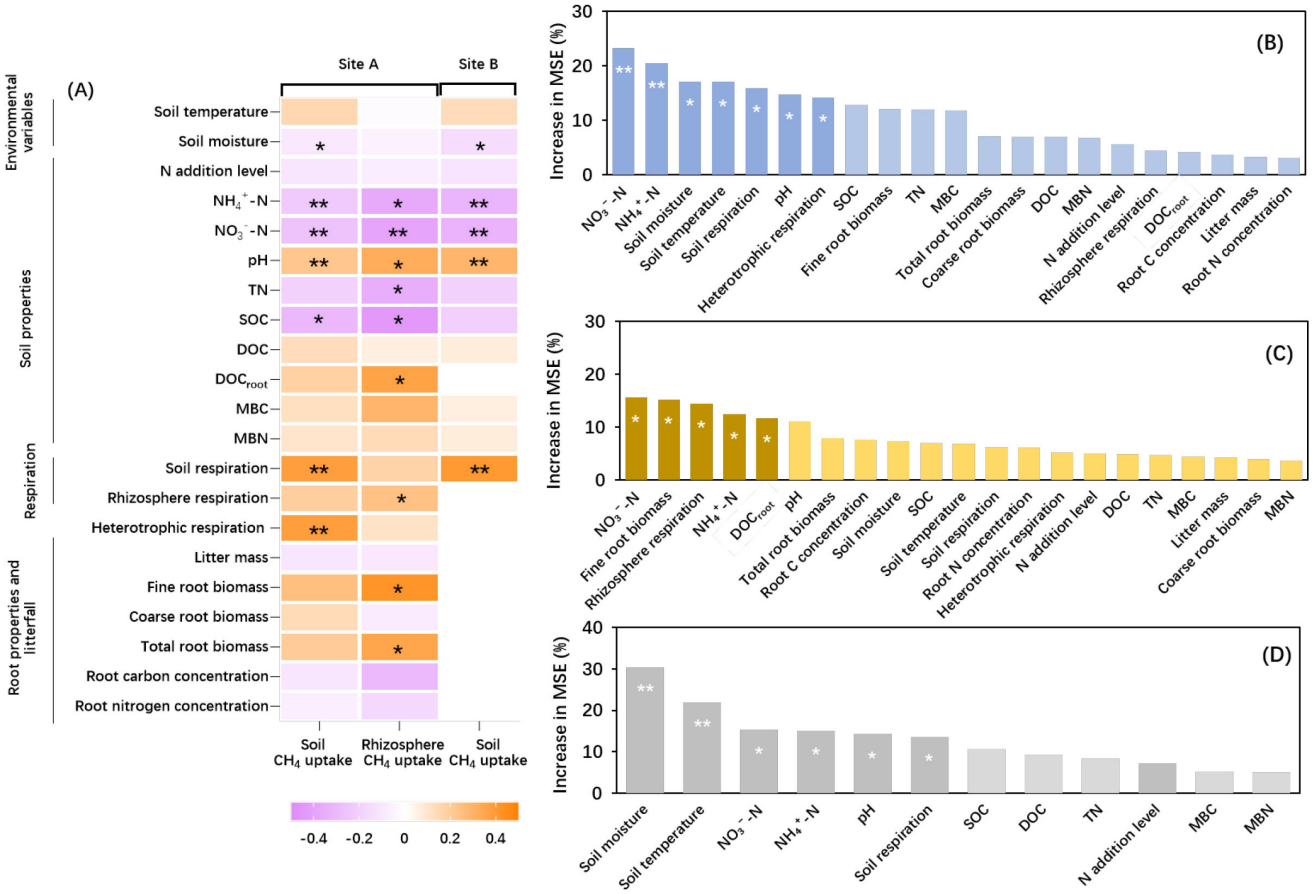
Regression coefficients of soil properties, environmental variables, root properties and soil respiration with soil CH_4_ uptake (SR treatment, including roots and bulk soil) and rhizosphere CH_4_ uptake (R treatment, root-only) at site A and soil CH_4_ uptake (SR treatment, including roots and bulk soil) at site B **(A)**. Random forest analysis showing the mean predictor importance (% of increase in mean square error, increase in MSE) of environment variable as drivers for the soil CH_4_ uptake **(B)**, rhizosphere CH_4_ uptake **(C)** at site A and the soil CH_4_ uptake at site B **(D)**. MBC, microbial biomass C; MBN, microbial biomass N; SOC, soil organic carbon; DOC, dissolved organic carbon; DOC_root_, dissolved organic carbon in rhizosphere soil; TC, total carbon; TN, total nitrogen; NH_4_^+^-N, soil ammonium content, NO_3_^-^-N, soil nitrate content; Root C concentration, root carbon concentration; Root N concentration, root nitrogen concentration. The symbols * and ** indicate significant correlations at the 0.05 and 0.01 levels, respectively.

Our meta-analysis showed that N addition significantly decreased soil CH_4_ uptake by 20.3% (**Figure 3**). Specifically, the effects of N addition on CH_4_ varied significantly among N addition levels (Q_B_ = 37.76, *P*< 0.05), with 0–20 kg N ha^-1^ yr^-1^ having no significant effect, whereas higher N inputs significantly suppressed soil CH_4_ uptake, coinciding with a marked increase in soil inorganic N content. In contrast, the responses of soil CH_4_ uptake, soil inorganic N content and soil moisture did not differ significantly between short- and long-term N addition (**Figure 4**).

### N-induced rhizosphere effects on soil CH_4_ uptake

At site A, rhizosphere CH_4_ uptake (R treatment, root-only effects) averaged 0.34 ± 0.03 nmol m^-2^ s^-1^ with no apparent seasonal variation, accounting for 22.7% of soil CH_4_ uptake (SR treatment) under control treatment (**Table 2**). Both N20 and N50 significantly decreased rhizosphere CH_4_ uptake (R treatment) by 38.2% and 32.4%, respectively (*P*< 0.05). The reductions in rhizosphere CH_4_ uptake explained 76.5% and 23.6% of the decreases in soil CH_4_ uptake under N20 and N50, respectively (**Supplementary Table 3**).

### Linking biotic and abiotic factors to soil CH_4_ uptake

Regression analyses revealed significant correlations between CH_4_ uptake (SR and R treatment) and multiple biotic and abiotic variables (**Figure 4A**). At site A, soil CH_4_ uptake (SR treatment) showed significant correlations with soil properties (NH_4_^+^-N, NO_3_^-^-N, pH, TN, and SOC; *P*< 0.05), environmental variables (soil temperature and moisture; *P*< 0.05), soil and heterotrophic respiration (*P*< 0.05). Rhizosphere CH_4_ uptake (R treatment) was significantly associated with inorganic N, pH, rhizosphere DOC, rhizosphere respiration, fine root biomass, and root carbon concentration (*P*< 0.05), but not with environmental variables. At site B, there were significant correlations between soil CH_4_ uptake (SR treatment) and soil properties (NH_4_^+^-N, NO_3_^-^-N, and pH; *P*< 0.05), environmental variables (soil temperature and moisture; *P*< 0.05) and soil respiration (*P*< 0.05). Random forest analysis revealed that the response of soil CH_4_ uptake to N addition at site A and site B was best explained by soil properties (NH_4_^+^-N and NO_3_^-^-N), environmental variables (soil moisture) and soil respiration (**Figures 4B, D**). By contrast, the response of rhizosphere CH_4_ uptake was best explained by soil properties, root properties and rhizosphere respiration (**Figure 4C**).

**Figure 5:**
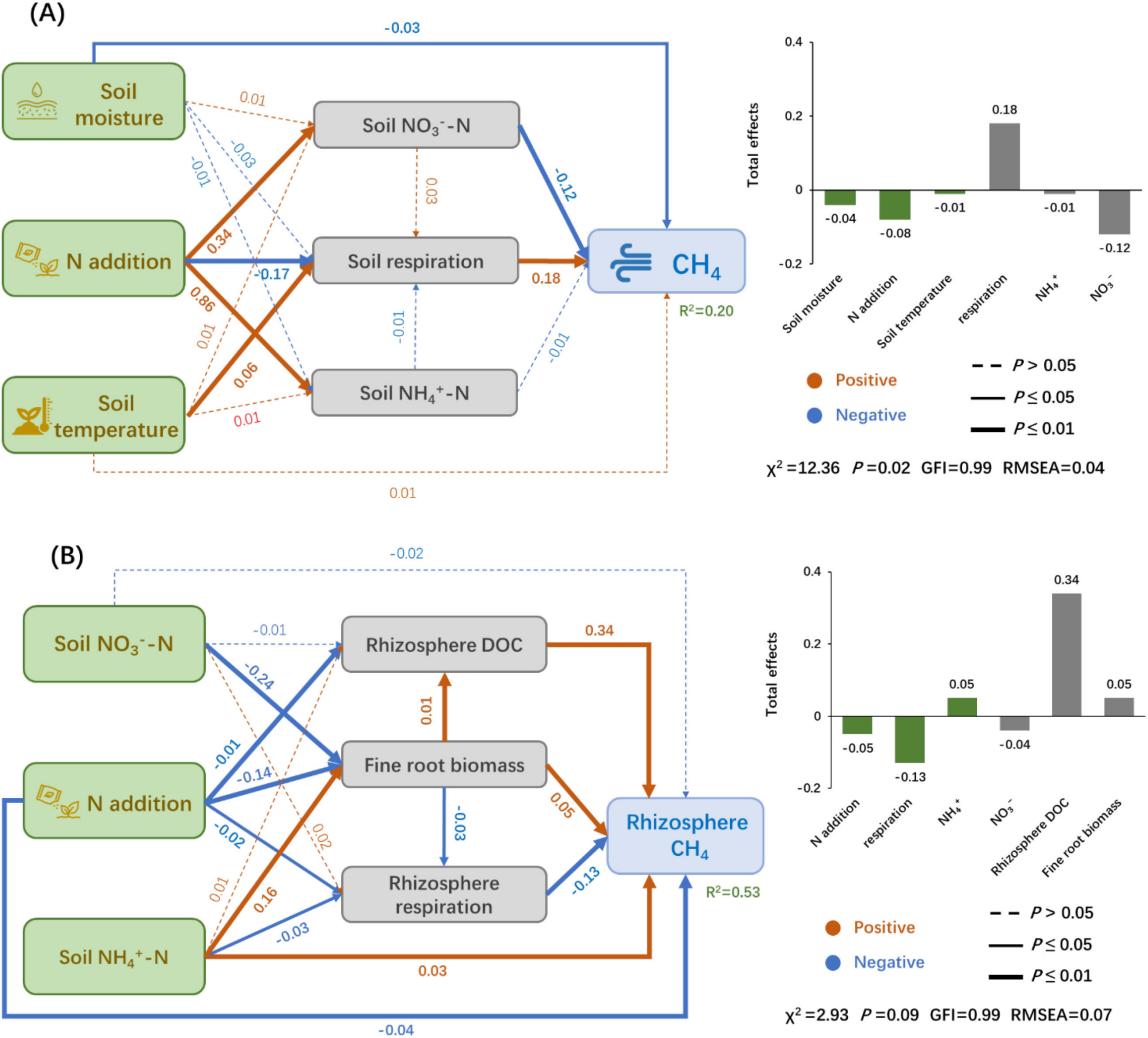
The interconnections across soil CH_4_ uptake (SR treatment) **(A)**, rhizosphere CH_4_ uptake (R treatment) **(B)** and all its controlling factors at site A, identified by SEM method. Orange and blue arrows indicate positive and negative relationships, respectively; bold and dash arrows indicate significant and non-significant relationships, respectively. The numbers on arrows are the standardized path coefficients and arrow width represented the strength of the relationship. Goodness-of-fit statistics are shown below the model.

The final SEMs explained 20% and 53% of the variation in soil CH_4_ uptake (SR treatment) and rhizosphere CH_4_ uptake (R treatment) at site A, respectively (**Figure 5**). For soil CH_4_ uptake, soil respiration exerted the strongest direct effect (*P*< 0.05), followed by NO_3_^-^-N, and N addition. Soil moisture, temperature, and N addition indirectly influenced soil CH_4_ uptake via their effects on soil respiration, NH_4_^+^-N, and NO_3_^-^-N (**Figure 5A**). For rhizosphere CH_4_ uptake, rhizosphere respiration consistently emerged as the dominant driver, followed by fine root biomass and rhizosphere DOC (**Figure 5B**).

## Discussion

**Figure 6:**
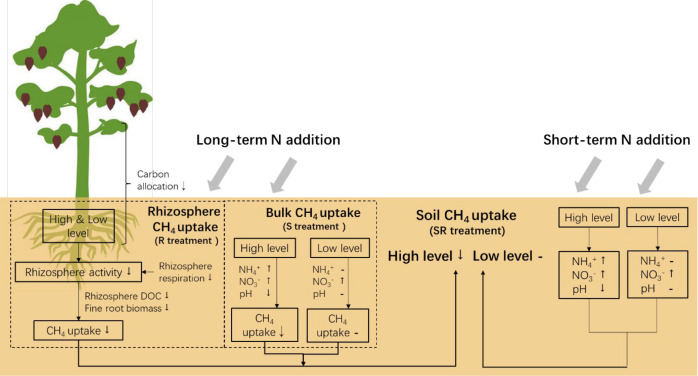
Graphical abstract for interpreting the N-induced rhizosphere effects and N addition duration effects on CH_4_ uptake (SR, R, and S treatment). The up and down arrows represent significant positive and negative responses (*P*< 0.05), respectively, and the straight short lines represent no significant responses.

Our field experiment was designed to evaluate the effects of N addition duration and N-induced rhizosphere processes on CH_4_ uptake (SR, R and S treatment). Based on our findings, we propose a conceptual model and discuss the potential underlying mechanisms in the following sections (**Figure 6**).

### Temporal variation of soil CH_4_ uptake at site A

At site A, soil CH_4_ uptake (SR treatment) ranged from approximately 1.35 to 1.44 nmol m^-2^ s^-1^ over the growing season, which was higher than values reported for temperate grassland (0.95 nmol m^-2^ s^-1^; Wang et al., 2019), but lower than those observed in undisturbed boreal forests (2.11 nmol m^-2^ s^-1^; Geng et al., 2017). These results suggest that soil CH_4_ uptake in plantations represents an intermediate level between grasslands and natural forests, highlighting the need for global process-based models to distinguish plantations from natural forests when estimating regional and global CH_4_ budgets.

Soil CH_4_ uptake (SR treatment) showed significant interannual variation (*P*< 0.05; **Figure 1B**), with the highest uptake observed in 2019. This increase was likely associated with lower soil moisture during that year (**Supplementary Figure 2**). Correlation analyses revealed a significant negative relationship between CH_4_ uptake and soil moisture and random forest analysis further indicated that soil moisture was as one of the dominant drivers for soil CH_4_ uptake (**Figure 4**) (Chen et al., 2019; Gao et al., 2023). Lower soil moisture content enhances O_2_ availability and accelerates CH_4_ diffusion into the soil, providing a favorable condition for methanotrophs (Bender and Conrad, 1992). Furthermore, the soil type at the study site was predominantly sandy, which maintains poor water-holding capacity (Ma et al., 2014) and contributed to overall low soil moisture levels during the growing season. However, temporal fluctuations in precipitation and other weather conditions caused notable variability in soil CH_4_ uptake (e.g., July 2018).

Soil CH_4_ uptake (SR treatment) was also positively correlated with soil temperature (**Figure 4**), in line with previous observations (Aronson et al., 2013; Zhu et al., 2015). Nonetheless, because soil temperature showed little variation among years, it likely played a minor role in driving the observed interannual variability in CH_4_ uptake.

### The contribution of rhizosphere on soil CH_4_ uptake

In our study, the rhizosphere accounted for 22.7% of total CH_4_ uptake at site A (**Table 2**). Comparisons with other ecosystems are limited due to the scarcity of published data. Subke et al. (2018) reported that the rhizosphere contributed 63.9% of soil CH_4_ uptake in a temperate forest, whereas Yue et al. (2021) found that the arbuscular mycorrhizal fungi contributed 15% to soil CH_4_ uptake in an ephemeral plant-dominated desert. Additionally, laboratory incubations in *Pinus tabulaeformis* forests showed higher CH_4_ uptake in rhizosphere soil than in bulk soil (Jing et al., 2021).

We found that rhizosphere CH_4_ uptake (R treatment) showed weak correlations with environmental variables but was strongly associated with soil NO_3_^-^, NH_4_^+^, root properties and rhizosphere respiration (**Figure 4**), suggesting that rhizosphere CH_4_ uptake was mainly regulated by rhizosphere activity (Chen et al., 2019, 2023).

Structural equation modeling (SEM) revealed that fine root biomass, rhizosphere respiration and rhizosphere DOC exerted a significant positive effect on rhizosphere CH_4_ uptake (standardized path coefficients = 0.15, 0.11 and 0.10, respectively; *p*< 0.05). Both rhizosphere respiration and DOC represent the metabolic activity of roots and their contribution to soil C substrates through root exudation and their contribution to soil C substrates through root exudation (Reich et al., 2008), which sustain methanotrophic activity in the rhizosphere (Subke et al., 2018). Li et al. (2022) showed that dissolved organic carbon and oxygen concentration in rhizosphere soil simultaneously promote methanotrophs activity and *pmoA* gene abundance. Moreover, roots and mycorrhizal fungi may enhance soil pore connectivity through the formation of soil aggregates, thereby increasing CH_4_ availability for methanotrophs (Lu et al., 2021; Six et al., 2006). In addition, roots and mycorrhizal fungi could provide labile carbon and nutrients that support methanotroph abundance and activity (Bodelier and Laanbroek, 2004; Praeg et al., 2016). In addition, plants colonized extensively by arbuscular mycorrhizal fungi support higher potential nitrification rates, which lead to lower soil ammonium content and thus favor methanotrophs (Veresoglou et al., 2019).

### Effects of N addition on soil CH_4_ uptake

After the three-year field experiment, we found that low-level N addition (N20) had no significant effect on soil CH_4_ uptake (SR treatment) (**Table 2**). This finding aligns with previous observations in temperate needle–broadleaved mixed forests, where similar N addition rates did not alter soil CH_4_ uptake (Geng et al., 2017). A recent meta-analysis further demonstrated a unimodal response of soil CH_4_ uptake to N addition, with low-level N addition stimulating CH_4_ oxidation in N-limited soils (Peng et al., 2019). Although we did not detect a net increase in mean CH_4_ uptake under N addition over three years, transient stimulation occurred on several individual sampling dates (**Supplementary Figure 1**). Soil NH_4_^+^-N and pH remained unchanged, while soil NO_3_^-^-N significantly accumulated under N20 (**Table 1** and **Supplementary Table 4**), likely due to the oxidation of NH_4_^+^-N assimilated by plants and microbes (Liu et al., 2017). Our meta-analysis further confirmed that N additions below 25 kg N ha^-1^ yr^-1^ had no significant impact on CH_4_ uptake in temperate forests (**Figure 5A**). In N-limited soils, low-level N additions may alleviate the N limitation of methanotrophic community growth or promote the synthesis of CH_4_-oxidizing enzymes (Bodelier and Laanbroek, 2004; Chen et al., 2023; Ma et al., 2020). Additionally, NO_3_^-^-N enrichment may enhance CH_4_ uptake when microbes experience N starvation within a specific range (Fender et al., 2012; Li et al., 2015).

In contrast, high-level N addition significantly suppressed soil CH_4_ uptake (SR treatment) at site A (**Figure 3A**). This pattern was consistent with our meta-analysis, which showed a transition from neutral to negative effects on CH_4_ uptake at around 25 kg N ha^-1^ yr^-1^. The decrease in soil CH_4_ uptake was mainly due to the increase in soil inorganic N content and microbial biomass C:N ratios (**Figure 4**). Elevated NH_4_^+^-N concentrations may competitively inhibit particulate methane monooxygenase (*pMMO*), reducing methanotrophic activity; moreover, excessive NO_3_^-^-N may exert direct toxic effects on methanotrophs (Chen et al., 2019; Gulledge et al., 2004; Walkiewicz and Brzezińska, 2019). Moreover, the higher microbial biomass C:N ratio.

observed under high-level N addition suggests intensified microbial N demand, increasing competition between methanotrophs and other microbial groups for available N, thereby further limiting CH_4_ uptake (Chen et al., 2023). In addition, nitrification intermediates such as hydroxylamine (NH_2_OH) and nitrite (NO_2_^-^) may be toxic to methanotrophs by destroying relevant enzymes and membranes (Bradford et al., 2001; Hu and Lu, 2015).

Unlike N addition level, N addition duration did not significantly influence soil CH_4_ uptake (SR treatment), consistent with our meta-analysis. While previous studies suggested that prolonged N addition could exacerbate CH_4_ uptake suppression through cumulative increases in soil inorganic N and acidification (Aronson and Helliker, 2010), such effects were not evident in our study. The concentrations of NH_4_^+^-N, NO_3_^--^N, and soil pH did not differ significantly between sites exposed to long- and short-term high-level N additions (**Table 2**). One possible explanation is that methanotrophic communities may undergo adaptive shifts under chronic N enrichment, thereby mitigating the inhibitory effects of sustained N loading (Jang et al., 2011).

### Rhizosphere-mediated effects of N addition on soil CH_4_ uptake

Our results showed that N addition significantly reduced rhizosphere CH_4_ uptake (R treatment) but had no significant effect on bulk CH_4_ uptake (S treatment) under low-level N addition at site A (**Figure 3C**). This suggests that the overall N-induced reduction in soil CH_4_ (SR treatment) may be largely attributed to decreased rhizosphere CH_4_ oxidation within the rhizosphere. These findings contrast with previous research conducted in rice paddies, where N fertilization stimulated root growth and rhizosphere CH_4_ oxidation, thereby mitigating CH_4_ emissions (Li et al., 2022). Such discrepancies likely arise because our study site is strongly N-limited.

In N-limited ecosystems, roots and mycorrhizal communities are highly sensitive to external N inputs (Yan et al., 2019). We observed significant differences in fine root biomass, rhizosphere respiration, and rhizosphere DOC between N addition and control plots (**Table 1**). According to the functional equilibrium hypothesis, trees allocate a substantial fraction of photosynthates to roots and mycorrhizal fungi to enhance N acquisition under N limitation (Janssens et al., 2010). When N limitation is alleviated by N fertilization, carbon allocation from plants to the rhizosphere may be reduced and suppress fine root activity (Sun et al., 2014). Root dynamics had significantly influenced methanotrophs. Consequently, the observed declines in rhizosphere respiration and DOC under N addition likely suppressed methanotrophic activity in the rhizosphere, leading to decreased rhizosphere CH_4_ uptake. Moreover, SEM results indicated that N-induced changes in fine root biomass affected soil NH_4_^+^-N and NO_3_^-^-N. This finding is consistent with Shen et al. (2023), who reported significantly higher NO_3_^-^-N concentrations following root exclusion in a detrital input and removal treatment (DIRT). Plants primarily use NH_4_^+^-N and NO_3_^-^-N as N sources (Gurmesa et al., 2022). Thus, increased soil N availability may reduce plant N demand and suppress fine root activity, thereby decreasing plant N uptake and leading to the accumulation of inorganic N, indirectly affecting rhizosphere CH_4_ uptake.

Overall, our findings emphasize that N addition exerts a strong negative influence on rhizosphere CH_4_ uptake by altering root dynamics and belowground C allocation. However, the responses of root exudates to varying N addition levels remain unclear, as do the specific exudate compounds that regulate methanotrophic activity. Furthermore, the linkages between rhizosphere CH_4_ uptake and fine root morphological traits are not yet fully understood. Future research should therefore explore a broader spectrum of belowground carbon fluxes to improve our understanding of how chronic N enrichment modifies soil N availability and rhizosphere CH_4_ dynamics.

## Conclusions

Our findings from a three-year field experiment and meta-analysis demonstrated that both rhizosphere activity and nitrogen (N) addition levels play crucial roles in regulating soil CH_4_ uptake in temperate forests. Rhizosphere processes accounted for approximately 22.7% of soil CH_4_ uptake, and N-induced suppression of root activity (e.g. fine root biomass, rhizosphere respiration and rhizosphere DOC) substantially reduced rhizosphere CH_4_ uptake. Low-level N addition had little effect on soil CH_4_ uptake, likely due to the alleviation of N limitation for methanotrophs and plants. In contrast, high-level N addition significantly decreased soil CH_4_ uptake, mainly through the accumulation of soil NH_4_^+^-N and NO_3_^-^-N. Overall, this study highlights the importance of root–microbe interactions in mediating CH_4_ dynamics under N enrichment. Future research should integrate continuous belowground C and N flux measurements with molecular and isotopic approaches to elucidate the mechanisms linking rhizosphere processes and CH_4_ oxidation under chronic N deposition.

## Data Availability

The original contributions presented in the study are included in the article/**Supplementary Material**. Further inquiries can be directed to the corresponding author.

## References

[B1] Aronson E. L. Allison S. D. Helliker B. R. (2013). Environmental impacts on the diversity of methane-cycling microbes and their resultant function. Front. Microbiol. 4. doi: 10.3389/fmicb.2013.00225. PMID: 23966984 PMC3743065

[B2] Ackerman D. Millet D. B. Chen X . (2019). Global estimates of inorganic nitrogen deposition across four decades. Global Biogeochem. Cy. 33, 100–107. doi: 10.1029/2018gb005990, PMID: 40890438

[B3] Aronson E. L. Helliker B. R. (2010). Methane flux in non-wetland soils in response to nitrogen addition a meta-analysis. Ecology 91, 3242–3251. doi: 10.1890/09-2185.1. PMID: 21141185

[B4] Bender M. Conrad R. (1992). Kinetics of CH_4_ oxidation in oxic soils exposed to ambient air or high CH_4_ mixing ratios. FEMS Microbiol. Ecol. 101, 261–270. doi: 10.1111/j.1574-6968.1992.tb05783.x. PMID: 41875165

[B5] Bodelier P. L. E. Laanbroek H. J. (2004). Nitrogen as a regulatory factor of methane oxidation in soils and sediments. FEMS Microbiol. Ecol. 47, 265–277. doi: 10.1016/S0168-6496(03)00304-0. PMID: 19712315

[B6] Bradford M. A. Wookey P. A. Ineson P. Lappin-Scott H. M . (2001). Controlling factors and effects of chronic nitrogen and sulphur deposition on methane oxidation in a temperate forest soil. Soil Biol. Biochem. 33, 93–102. doi: 10.1016/S0038-0717(00)00118-8, PMID: 41334505

[B7] Brookes P. C. Landman A. Pruden G. Jenkinson D. S. (1985). Chloroform fumigation and the release of soil nitrogen, a rapid direct extraction method to measure microbial biomass nitrogen in soil. Soil Biol. Biochem. 17, 837–842. doi: 10.1016/0038-0717(85)90144-0

[B8] Chan A. S. K. Steudler P. A. Bowden R. D. Gulledge J. Cavanaugh C. M. (2005). Consequences of nitrogen fertilization on soil methane consumption in a productive temperate deciduous forest. Biol. Fert. Soils 41, 182–189. doi: 10.1007/s00374-004-0822-7. PMID: 41894112

[B9] Chen X. Diao H. Wang S. Li H. Wang Z. Shen Y. . (2023). Plant community mediated methane uptake in response to increasing nitrogen addition level in a saline-alkaline grassland by rhizospheric effects. Geoderma 429, 116235. doi: 10.1016/j.geoderma.2022.116235. PMID: 41903563

[B10] Chen S. Hao T. Goulding K. Misselbrook T. Liu X. (2019). Impact of 13-years of nitrogen addition on nitrous oxide and methane fluxes and ecosystem respiration in a temperate grassland. Environ. pollut. 252, 675–681. doi: 10.1016/j.envpol.2019.03.069. PMID: 31185356

[B11] Chinese Ministry of Forestry (2014). Forest resource statistics of China. Department of forest resource and management (Beijing, China: Chinese Ministry of Forestry).

[B12] Cui Y. X. Peng S. S. Rillig M. C. Camenzind T. Delgado-Baquerizo M. Terrer C. . (2025). Global patterns of nutrient limitation in soil microorganisms. Proc. Natl. Acad. Sci. 122, e2424552122. doi: 10.1073/pnas.2424552122. PMID: 40359040 PMC12107151

[B13] Du E. Z. Fang J. (2014). Linking belowground and aboveground phenology in two boreal forests in Northeast China. Oecologia 176, 883–892. doi: 10.1007/s00442-014-3055-y. PMID: 25164492

[B14] Etminan M. Myhre G. Highwood E. J. Shine K. P. (2016). Radiative forcing of carbon dioxide, methane, and nitrous oxide, A significant revision of the methane radiative forcing. Geophys. Res. Lett. 43, 12614–12623. doi: 10.1002/2016gl071930. PMID: 41889077

[B15] Fender A. C. Pfeiffer B. Gansert D. Leuschner C. Daniel R. Jungkunst H. F. (2012). The inhibiting effect of nitrate fertilisation on methane uptake of a temperate forest soil is influenced by labile carbon. Biol. Fert. Soils 48, 621–631. doi: 10.1007/s00374-011-0660-3. PMID: 41894112

[B16] Fransson P. Andersson A. Norstrom S. Bylund D. Bent E. (2016). Ectomycorrhizal exudates and pre-exposure to elevated CO_2_ affects soil bacterial growth and community structure. Funct. Ecol. 20, 211–224. doi: 10.1016/j.funeco.2016.01.003. PMID: 41903563

[B17] Gao W. Yang X. Zhang Y. Zhao T. Shi B. Yang T. . (2023). Suppression of methane uptake by precipitation pulses and long-term nitrogen addition in a semi-arid meadow steppe in northeast China. Front. Plant Sci. 13. doi: 10.3389/fpls.2022.1071511. PMID: 36726673 PMC9884686

[B18] Geng J. Cheng S. Fang H. Yu G. Li X. Si G. . (2017). Soil nitrate accumulation explains the nonlinear responses of soil CO_2_ and CH_4_ fluxes to nitrogen addition in a temperate needle-broadleaved mixed forest. Ecol. Ind. 79, 28–36. doi: 10.1016/j.ecolind.2017.03.054. PMID: 41903563

[B19] Gong Y. Wu J. Le T. B. (2021). Counteractions between biotic and abiotic factors on methane dynamics in a boreal peatland, vegetation composition change vs warming and nitrogen deposition. Geoderma 395, 115074. doi: 10.1016/j.geoderma.2021.115074. PMID: 41903563

[B20] Gulledge J. Hrywna Y. Cavanaugh C. Steudler P. A. (2004). Effects of long-term nitrogen fertilization on the uptake kinetics of atmospheric methane in temperate forest soils. FEMS Microbiol. Ecol. 49, 389–400. doi: 10.1016/j.femsec.2004.04.013. PMID: 19712289

[B21] Gurmesa G. A. Wang A. Li S. Peng S. de Vries W. Gundersen P. . (2022). Retention of deposited ammonium and nitrate and its impact on the global forest carbon sink. Nat. Commun. 13, 880. doi: 10.1038/s41467-022-28345-1. PMID: 35169118 PMC8847626

[B22] Hedges L. V. Gurevitch J. Curtis P. S. (1999). The meta-analysis of response ratios in experimental ecology. Ecology 80, 1150–1156. doi: 10.2307/177062

[B23] Hogberg P. Nordgren A. Buchmann N. Taylor A. F. S. Ekblad A. Hogberg M. N. . (2001). Large-scale forest girdling shows that current photosynthesis drives soil respiration. Nature 411, 789–793. doi: 10.1038/35081058. PMID: 11459055

[B24] Hu A. Lu Y. (2015). The differential effects of ammonium and nitrate on methanotrophs in rice field soil. Soil Biol. Biochem. 85, 31–38. doi: 10.1016/j.soilbio.2015.02.033. PMID: 41903563

[B25] Jang I. Lee S. Zoh K. D. Kang H. (2011). Methane concentrations and methanotrophic community structure influence the response of soil methane oxidation to nitrogen content in a temperate forest. Soil Biol. Biochem. 43, 620–627. doi: 10.1016/j.soilbio.2010.11.032. PMID: 41903563

[B26] Janssens I. A. Dieleman W. Luyssaert S. Subke J. A. Reichstein M. Ceulemans R. . (2010). Reduction of forest soil respiration in response to nitrogen deposition. Nat. Geosci. 3, 315–322. doi: 10.1038/ngeo844. PMID: 41896565

[B27] Jing H. Liu Y. Wang G. L. Liu G. B. (2021). Contrasting effects of nitrogen addition on rhizosphere soil CO_2_, N_2_O, and CH_4_ emissions of fine roots with different diameters from Pinus tabulaeformis forest using laboratory incubation. Sci. Tot. Environ. 780, 146298. doi: 10.1016/j.scitotenv.2021.146298. PMID: 33770604

[B28] Li X. Cheng S. Fang H. Yu G. Dang X. Xu M. . (2015). The contrasting effects of deposited NH_4_^+^ and NO_3_^-^ on soil CO_2_, CH_4_ and N_2_O fluxes in a subtropical plantation, southern China. Ecol. Eng. 85, 317–327. doi: 10.1016/j.ecoleng.2015.10.003. PMID: 41903563

[B29] Li Q. Cui K. Lv J. Zhang J. Peng C. Li Y. . (2022). Biochar amendments increase soil organic carbon storage and decrease global warming potentials of soil CH_4_ and N_2_O under N addition in a subtropical Moso bamboo plantation. For. Ecosyst. 9, 10.1016/j.fecs.2022.100054. doi: 10.1016/j.fecs.2022.100054. PMID: 41903563

[B30] Liu L. Greaver T. L. (2009). A review of nitrogen enrichment effects on three biogenic GHGs, the CO_2_ sink may be largely offset by stimulated N_2_O and CH_4_ emission. Ecol. Lett. 12, 1103–1117. doi: 10.1111/j.1461-0248.2009.01351.x. PMID: 19694782

[B31] Liu X. Zhang Q. Li S. Zhang L. Ren J. (2017). Simulated NH_4_^+^-N deposition inhibits CH_4_ uptake and promotes N_2_O emission at the meadow steppe of Inner Mongolia, China. Pedosphere 27, 306–317. doi: 10.1016/s1002-0160(17)60318-7. PMID: 41867250

[B32] Lu X. Hou E. Guo J. Gilliam F. S. Li J. Tang S. . (2021). Nitrogen addition stimulates soil aggregation and enhances carbon storage in terrestrial ecosystems of China, A meta-analysis. Glob. Change Biol. 00, 1–13. doi: 10.1111/gcb.15604. PMID: 33742519

[B33] Ma Y. Piao S. L. Sun Z. Lin X. Wang T. Yue C. . (2014). Stand ages regulate the response of soil respiration to temperature in a Larix principis-rupprechtii plantation. Agr. For. Meteorol. 184, 179–187. doi: 10.1016/j.scitotenv.2019.136048. PMID: 31864135

[B34] Ma L. Yang H. Pan Z. Rong Y. (2020). In situ measurements and meta-analysis reveal that land-use changes combined with low nitrogen application promote methane uptake by temperate grasslands in China. Sci. Tot. Environ. 706, 136048. doi: 10.1016/j.scitotenv.2019.136048. PMID: 31864135

[B35] Maier M. Paulus S. Nicolai C. Stutz K. P. Nauer P. A. (2017). Drivers of site-Scale variability of CH_4_ consumption in a well-aerated pine forest soil. Forests 8, 12–17. doi: 10.3390/f8060193, PMID: 41725453

[B36] Murguia-Flores F. Arndt S. Ganesan A. L. Murray-Tortarolo G. Hornibrook E. R. C. (2018). Soil methanotrophy model (MeMo v1.0), a process-based model to quantify global uptake of atmospheric methane by soil. Geosci. Model. Dev. 11, 2009–2032. doi: 10.5194/gmd-11-2009-2018. PMID: 41899199

[B37] Nyerges G. Stein L. Y. (2009). Ammonia cometabolism and product inhibition vary considerably among species of methanotrophic bacteria. FEMS Microbiol. Lett. 297, 131–136. doi: 10.1111/j.1574-6968.2009.01674.x. PMID: 19566684

[B38] Payn T. Carnus J. M. Freer-Smith P. Kimberley M. Kollert W. Liu S. . (2015). Changes in planted forests and future global implications. For. Ecol. Manage. 352, 57–67. doi: 10.1016/j.foreco.2015.06.021. PMID: 41903563

[B39] Peng Y. Wang G. Li F. Yang G. Fang K. Liu L. . (2019). Unimodal response of soil methane consumption to increasing nitrogen additions. Environ. Sci. Technol. 53, 4150–4160. doi: 10.1021/acs.est.8b04561. PMID: 30892031

[B40] Praeg N. Wagner A. O. Illmer P. (2016). Plant species, temperature, and bedrock affect net methane flux out of grassland and forest soils. Plant Soil 410, 193–206. doi: 10.1007/s11104-016-2993-z. PMID: 41894112

[B41] Reich P. B. Tjoelker M. G. Pregitzer K. S. Wright I. J. Oleksyn J. ManChado J. L. (2008). Scaling of respiration to nitrogen in leaves, stems and roots of higher land plants. Ecol. Lett. 11, 793–801. doi: 10.1111/j.1461-0248.2008.01185.x. PMID: 18445031

[B42] Saunois M. Stavert A. R. Poulter B. Bousquet P. Canadell J. G. Jackson R. B. . (2020). The global methane budget 2000-2017. Earth Syst. Sci. Data 12, 1561–1623. doi: 10.5194/essd-12-1561-2020. PMID: 41899199

[B43] Shah J. A. Yue C. Xiong Y. Lin N. Wu J. (2025). Grass-legume polyculture enhances plant productivity and nutrient availability by modulating soil enzymatic activities and microbial communities in grassland. J. Soil. Sediment. 25, 2837–2853. doi: 10.1007/s11368-025-04126-3. PMID: 41894112

[B44] Shen Y. Feng J. Zhou D. He K. Zhu B . (2023). Impacts of aboveground litter and belowground roots on soil greenhouse gas emissions: Evidence from a DIRT experiment in a pine plantation. Agr. For. Meteorol. 343, 109792. doi: 10.1016/j.agrformet.2023.109792, PMID: 41936479

[B45] Six J. Frey S. D. Thiet R. K. Batten K. M. (2006). Bacterial and fungal contributions to carbon sequestration in agroecosystems. Soil Sci. Soc Am. J. 70, 555–569. doi: 10.2136/sssaj2004.0347

[B46] Stiles W. A. V. Rowe E. C. Dennis P. (2018). Nitrogen and phosphorus enrichment effects on CO_2_ and methane fluxes from an upland ecosystem. Sci. Tot. Environ. 618, 1199–1209. doi: 10.1016/j.scitotenv.2017.09.202. PMID: 28954703

[B47] Subke J. A. Moody C. S. Hill T. C. Voke N. Toet S. Ineson P. . (2018). Rhizosphere activity and atmospheric methane concentrations drive variations of methane fluxes in a temperate forest soil. Soil Biol. Biochem. 116, 323–332. doi: 10.1016/j.soilbio.2017.10.037. PMID: 41903563

[B48] Sullivan B. W. Selmants P. C. Hart S. C. (2013). Does dissolved organic carbon regulate biological methane oxidation in semiarid soils? Glob. Change Biol. 19, 2149–2157. doi: 10.1111/gcb.12201. PMID: 23526765

[B49] Sun L. Ataka M. Han M. Han Y. Gan D. Xu T. . (2021). Root exudation as a major competitive fine-root functional trait of 18 coexisting species in a subtropical forest. New Phytol. 229, 259–271. doi: 10.1111/nph.16865. PMID: 32772392

[B50] Sun Z. Liu L. Ma Y. Yin G. Zhao C. Zhang Y. . (2014). The effect of nitrogen addition on soil respiration from a nitroge-limited forest soil. Agr. For. Meteorol. 197, 103–110. doi: 10.1016/j.agrformet.2014.06.010. PMID: 41903563

[B51] Sun Z. Liu L. Peng S. Peñuelas J. Zeng H. Piao S. (2016). Age-related modulation of the nitrogen resorption efficiency response to growth requirements and soil nitrogen availability in a temperate pine plantation. Ecosystems 19, 698–709. doi: 10.1007/s10021-016-9962-5. PMID: 41894112

[B52] Tamale J. Hueppi R. Griepentrog M. Turyagyenda L. F. Barthel M. Doetterl S. . (2021). Nutrient limitations regulate soil greenhouse gas fluxes from tropical forests, evidence from an ecosystem-scale nutrient manipulation experiment in Uganda. Soil 7, 433–451. doi: 10.5194/soil-7-433-2021. PMID: 41899199

[B53] Vance E. D. Brookes P. C. Jenkinson D. S. (1987). An extraction method for measuring soil microbial biomass C. Soil Biol. Biochem. 19, 703–707. doi: 10.1016/0038-0717(87)90052-6

[B54] Veresoglou S. D. Verbruggen E. Makarova O. Mansour I. Sen R. Rillig M. C. (2019). Arbuscular mycorrhizal fungi alter the community structure of ammonia oxidizers at high fertility via competition for soil NH_4_^+^. Microbiol. Ecol. 78, 147–158. doi: 10.1007/s00248-018-1281-2. PMID: 30402724

[B55] Walkiewicz A. Brzezińska M. (2019). Interactive effects of nitrate and oxygen on methane oxidation in three different soils. Soil Biol. Biochem. 133, 116–118. doi: 10.1016/j.envpol.2016.02.048. PMID: 26946175

[B56] Wang J. Hayes F. Chadwick D. R. Hill P. W. Mills G. Jones D. L. (2019). Short-term responses of greenhouse gas emissions and ecosystem carbon fluxes to elevated ozone and N fertilization in a temperate grassland. Atmos. Environ. 211, 204–213. doi: 10.1016/j.atmosenv.2019.05.027. PMID: 41903563

[B57] Wang W. Peng S. Wang T. Fang J. (2010). Winter soil CO_2_ efflux and its contribution to annual soil respiration in different ecosystems of a forest-steppe ecotone, North China. Soil Biol. Biochem. 42, 451–458. doi: 10.1016/j.soilbio.2009.11.028. PMID: 41903563

[B58] Wu J. Cheng X. Xing W. Liu G. (2022). Soil-atmosphere exchange of CH_4_ in response to nitrogen addition in diverse upland and wetland ecosystems, a meta-analysis. Soil Biol. Biochem. 164, 108467. doi: 10.1016/j.soilbio.2021.108467. PMID: 41903563

[B59] Xu M. Cheng S. Fang H. Yu G. Gao W. Wang Y. . (2014). Low-level nitrogen addition promotes net methane uptake in a boreal forest across the great Xing'an mountain region, China. For. Sci. 60, 973–981. doi: 10.5849/forsci.13-075

[B60] Yan T. Qu T. Song H. Sun Z. Zeng H. Peng S. S. (2019). Ectomycorrhizal fungi respiration quantification and drivers in three differently-aged larch plantations. Agr. For. Meteorol. 265, 245–251. doi: 10.1016/j.agrformet.2018.11.024. PMID: 41903563

[B61] Yan T. Qu T. Sun Z. Dybzinski R. Chen A. Yao X. . (2018). Negative effect of nitrogen addition on soil respiration dependent on stand age, evidence from a 7-year field study of larch plantations in northern China. Agr. For. Meteorol. 262, 24–33. doi: 10.1016/j.agrformet.2018.06.029. PMID: 41903563

[B62] Yue P. Cui X. Zuo X. Li K. Wang S. Jia Y. . (2021). The contribution of arbuscular mycorrhizal fungi to ecosystem respiration and methane flux in an ephemeral plants-dominated desert. Land. Degrad. Dev. 32, 1844–1853. doi: 10.1002/ldr.3838. PMID: 41889077

[B63] Zhu X. Luo C. Wang S. Zhang Z. Cui S. Bao X. . (2015). Effects of warming, grazing/cutting and nitrogen fertilization on greenhouse gas fluxes during growing seasons in an alpine meadow on the Tibetan Plateau. Agr. For. Meteorol. 214-215, 506–514. doi: 10.1016/j.agrformet.2015.09.008. PMID: 41903563

