# Supplementary material for: Nitrogen fertilization and rhizosphere processes regulate methane uptake in a nitrogen-limited forest
Source: Front Plant Sci. 2026 Apr 20;17:1719243. doi: 10.3389/fpls.2026.1719243 (PMC13137368; doi:10.3389/fpls.2026.1719243)
Supplement: Supplementary file 1 [file DataSheet1.docx]

**Supplementary materials of “N fertilization and rhizosphere activity drive variations of methane fluxes in a nitrogen-limited forest”**

Jianyu Chen^a^, Ying Deng^b*^

The following Supporting Information is available for this article:

**Figure S1** The distribution of the experimental sites and fluxes measured in each site.

**Figure S2** Temporal variation of soil temperature and soil moisture in (a) site A and (c) site B, and their annual means (b, d) for both sites.

**Figure S3** Temporal variation of CH4 uptake in larch plantations at site A from (a) SR treatment, (b) S treatment, (c) R treatment, (d) R/SR.

**Table S1** Summary of site A and site B stand properties of *Larix principis-rupprechtii* plantation in Saihanba.

**Table S2** Detailed site information for studies selected in our meta-analysis.

**Table S3** The reduction in CH4 uptake under N addition at site A.

**Table S4** Results (F value) of two-way ANOVAs on the effects of nitrogen additions (N), experimental year (Y), and their interactions (N × Y) effects on biotic and abiotic variables at site A and site B.


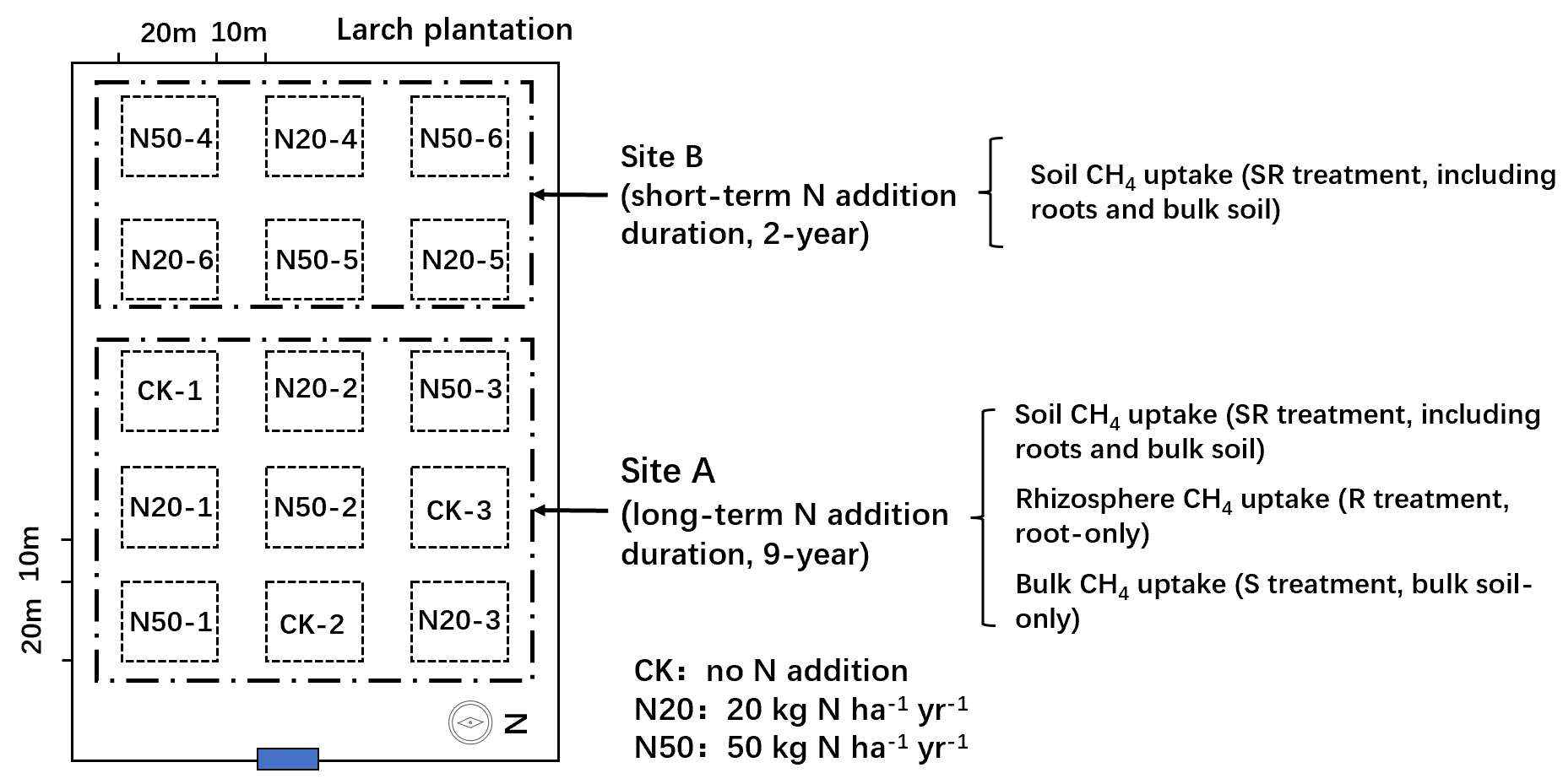


Figure S1 The distribution of the experimental sites and fluxes measured in each site


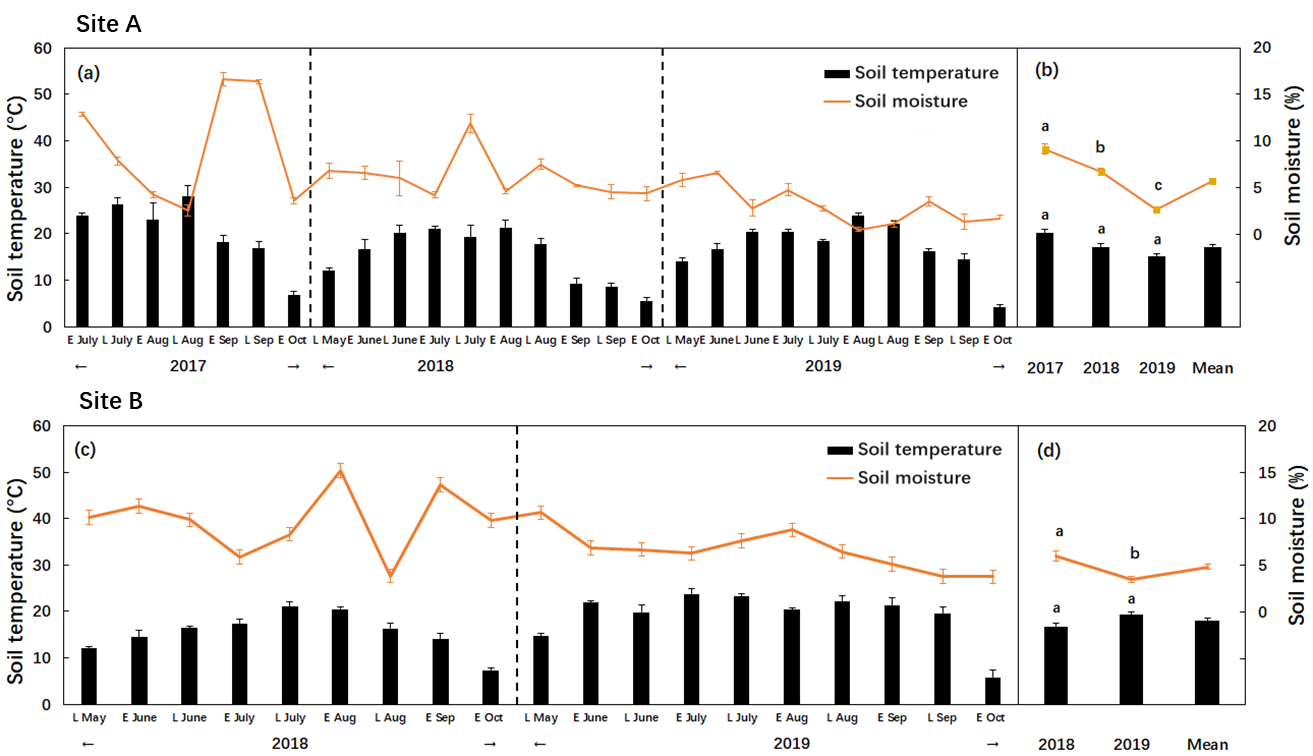


Figure S2 Temporal variation of soil temperature and soil moisture in (a) site A and (c) site B, and their annual means (b, d) for both sites. Different lowercase letters between different years denote significant differences (one-way ANOVA, post hoc LSD test, *P* < 0.05).


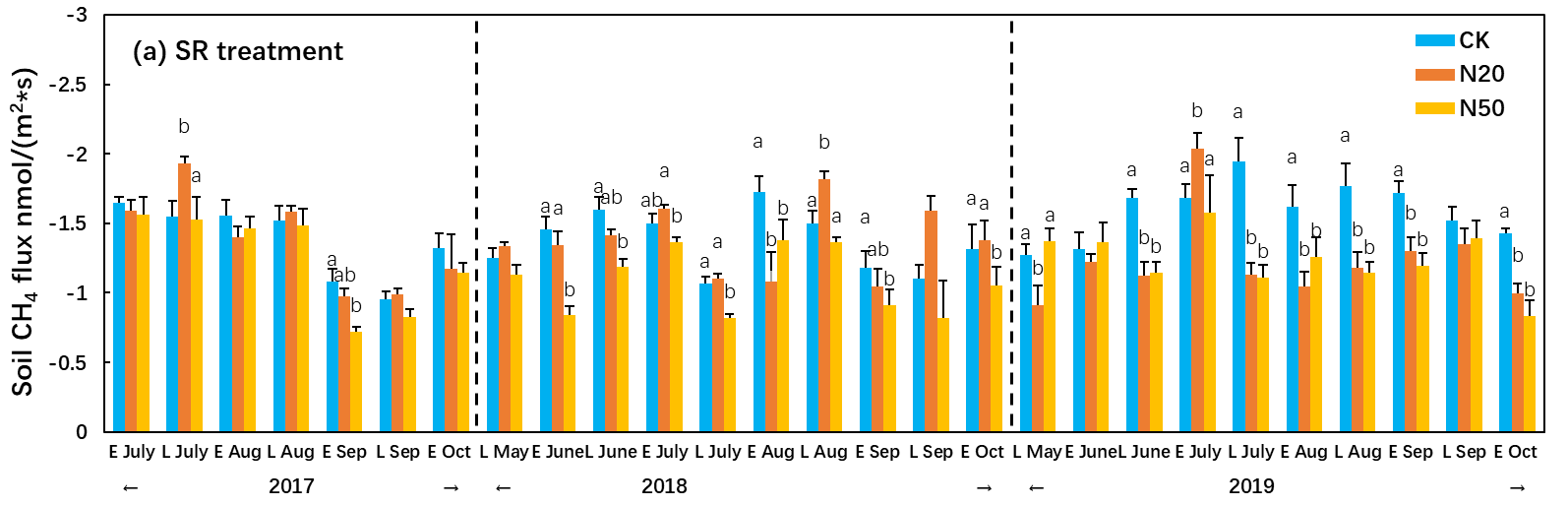

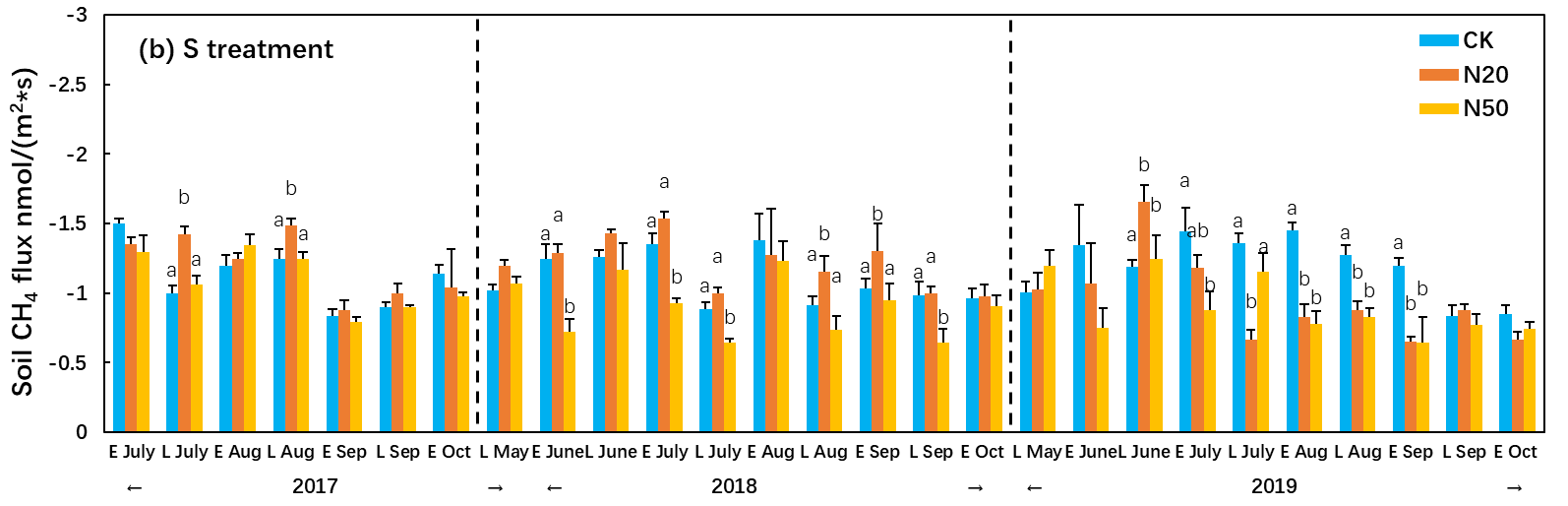

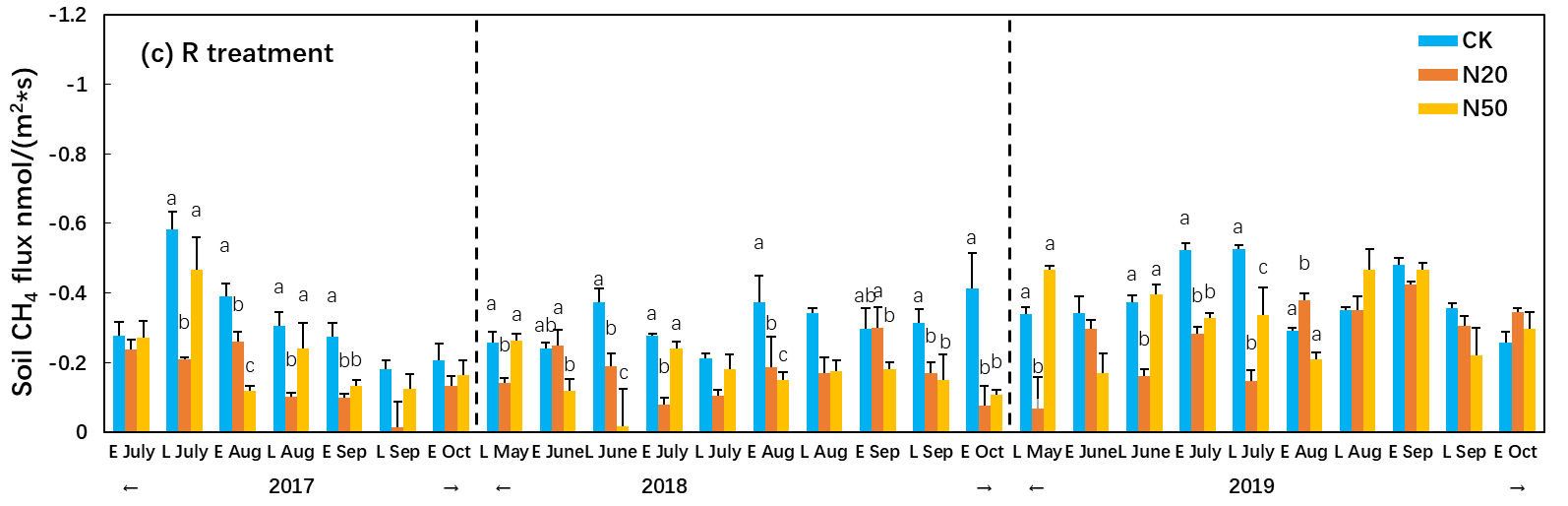

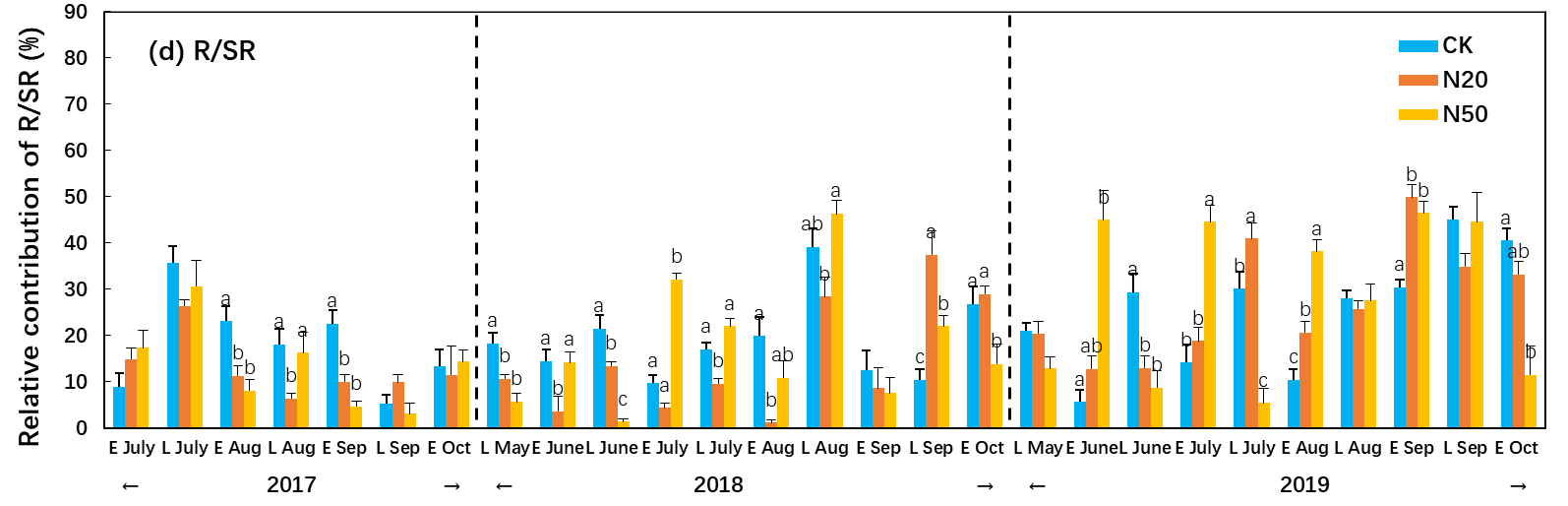


Figure S3 Temporal variation of CH_4_ uptake in larch plantations at site A from (a) SR treatment, (b) S treatment, (c) R treatment under N addition, (d) R/SR. CK, N20 and N50 represent 0, 20 and 50 Kg N ha^-1^ yr^-1^ N addition, respectively. Different letters (a, b, and c) between different N addition levels denote significant differences (one-way ANOVA, post hoc LSD test, *P* < 0.05).

Table S1 Summary of site A and site B stand properties of *Larix principis-rupprechtii* plantation in Saihanba

|  | Site A | Site B |
| --- | --- | --- |
| Stand density (tree ha^-1^) | 870 ± 48 | 820 ± 54 |
| DBH (m) | 19.9 ± 2.8 | 20.5 ± 5.5 |
| Basal area (m^2^ ha^-1^) | 48.4 ± 3.8 | 45.2 ± 3.7 |
| Tree Height (m) | 15.8 ± 1.6 | 16.0 ± 2.1 |
| Soil pH | 6.5 ± 0.2 | 6.6 ± 0.2 |
| Soil bulk density (g cm^-3^) | 1.47 | 1.51 |
| Soil texture (sand, silt, clay, %) | 74.0, 15.4, 10.6 | 77.3, 14.8, 7.9 |
| Understory species | Geum aleppicum Agrimonia Pilosa Sanguisorba tenuifolia | Geum aleppicum Agrimonia pilosa Sanguisorba tenuifolia |

Table S2 Detailed site information for studies selected in our meta-analysis. MAT = mean annual temperature (°C), MAP = mean annual precipitation (mm)

| Reference source | Forest type | Country | Longitude | Latitude | N addition duration | Stand age | Altitude | MAT | MAP | N addition type | N addition level | CK-methane flux (μg C m-2 h-1) | N-methane flux (μg C m-2 h-1) |
| --- | --- | --- | --- | --- | --- | --- | --- | --- | --- | --- | --- | --- | --- |
| Jassal et al., 2011 | Temperate forest | Canada | 49°52'7"N | 125°20'6"W | 1 | 60 |  | 8.6 | 1450 | Urea | 200 | -65.12 | -30.08 |
| Steudler et al., 1989 | Temperate forest | USA | 42°5′N | 72°00′W | 1 | 62 |  |  |  | NH_4_NO_3_ | 37 | -110.00 | -102.92 |
| Steudler et al., 1989 | Temperate forest | USA | 42°5′N | 72°00′W | 1 | 62 |  |  |  | NH_4_NO_3_ | 120 | -110.00 | -89.91 |
| Steudler et al., 1989 | Temperate forest | USA | 42°5′N | 72°00′W | 1 | 80 |  |  |  | NH_4_NO_3_ | 37 | -130.00 | -102.24 |
| Steudler et al., 1989 | Temperate forest | USA | 42°5′N | 72°00′W | 1 | 80 |  |  |  | NH_4_NO_3_ | 120 | -130.00 | -103.58 |
| Aronson et al., 2012 | Temperate forest | USA | 39°55′N | 74°35′W | 1 |  | 30 | 12.3 | 1143 | NH_4_NO_3_ | 5 | -40.48 | -45.78 |
| Aronson et al., 2012 | Temperate forest | USA | 39°55′N | 74°35′W | 1 |  | 30 | 12.3 | 1143 | NH_4_NO_3_ | 67 | -40.48 | -63.49 |
| Wang et al., 2016 | Temperate forest | China | 26°44′39″N | 115°03′33″E | 2 | 28 |  | 17.9 | 1505 | NH_4_Cl | 40 | -5.05 | -3.82 |
| Wang et al., 2016 | Temperate forest | China | 26°44′39″N | 115°03′33″E | 2 | 28 |  | 17.9 | 1505 | NH_4_Cl | 120 | -5.05 | -2.14 |
| Wang et al., 2016 | Temperate forest | China | 26°44′39″N | 115°03′33″E | 2 | 28 |  | 17.9 | 1505 | NaNO3 | 40 | -5.05 | -3.87 |
| Wang et al., 2016 | Temperate forest | China | 26°44′39″N | 115°03′33″E | 2 | 28 |  | 17.9 | 1505 | NaNO3 | 120 | -5.05 | -3.71 |
| Kim et al., 2012 | Temperate forest | Japan | 43°60′N | 141°20′E | 2 | 3 |  | 9.5 | 995 | NH_4_NO_3_ | 50 | -8.25 | -4.26 |
| Geng et al., 2017 | Temperate forest | China | 41°42′N | 127°38′E | 1 |  | 740 | 3.6 | 745 | Urea | 10 | -12.03 | -18.09 |
| Geng et al., 2017 | Temperate forest | China | 41°42′N | 127°38′E | 1 |  | 740 | 3.6 | 745 | Urea | 20 | -12.03 | -16.61 |
| Geng et al., 2017 | Temperate forest | China | 41°42′N | 127°38′E | 1 |  | 740 | 3.6 | 745 | Urea | 40 | -12.03 | -12.72 |
| Geng et al., 2017 | Temperate forest | China | 41°42′N | 127°38′E | 1 |  | 740 | 3.6 | 745 | Urea | 60 | -12.03 | -12.09 |
| Geng et al., 2017 | Temperate forest | China | 41°42′N | 127°38′E | 1 |  | 740 | 3.6 | 745 | Urea | 80 | -12.03 | -10.03 |
| Geng et al., 2017 | Temperate forest | China | 41°42′N | 127°38′E | 1 |  | 740 | 3.6 | 745 | Urea | 100 | -12.03 | -7.92 |
| Geng et al., 2017 | Temperate forest | China | 41°42′N | 127°38′E | 1 |  | 740 | 3.6 | 745 | Urea | 120 | -12.03 | -8.66 |
| Geng et al., 2017 | Temperate forest | China | 41°42′N | 127°38′E | 1 |  | 740 | 3.6 | 745 | Urea | 140 | -12.03 | -5.35 |
| Ambus et al., 2006 | Temperate forest | USA | 42°24'N | 85°24' W | 2 |  |  | 9.7 | 890 | NH_4_NO_3_ | 10 | -19.01 | -19.13 |
| Ambus et al., 2006 | Temperate forest | USA | 42°24'N | 85°24' W | 2 |  |  | 9.7 | 890 | NH_4_NO_3_ | 30 | -19.01 | -14.67 |
| Ambus et al., 2006 | Temperate forest | USA | 42°24'N | 85°24' W | 2 | 50 |  | 9.7 | 890 | NH_4_NO_3_ | 10 | -36.35 | -35.54 |
| Ambus et al., 2006 | Temperate forest | USA | 42°24'N | 85°24' W | 2 | 50 |  | 9.7 | 890 | NH_4_NO_3_ | 30 | -36.35 | -44.90 |
| Sitaula et al., 1995 | Temperate forest | Norway | 58°49' N | 8°32'E | 1 | 100 |  |  |  | NH_4_NO_3_ | 30 | -70.83 | -58.33 |
| Sitaula et al., 1995 | Temperate forest | Norway | 58°49' N | 8°32'E | 1 | 100 |  |  |  | NH_4_NO_3_ | 90 | -70.83 | -45.83 |
| Sitaula et al., 1995 | Temperate forest | Norway | 58°49' N | 8°32'E | 1 | 100 |  |  |  | NH_4_NO_3_ | 30 | -44.58 | -40.83 |
| Sitaula et al., 1995 | Temperate forest | Norway | 58°49' N | 8°32'E | 1 | 100 |  |  |  | NH_4_NO_3_ | 30 | -44.58 | -27.50 |
| Sitaula et al., 1995 | Temperate forest | Norway | 58°49' N | 8°32'E | 1 | 100 |  |  |  | NH_4_NO_3_ | 30 | -50.00 | -33.33 |
| Sitaula et al., 1995 | Temperate forest | Norway | 58°49' N | 8°32'E | 1 | 100 |  |  |  | NH_4_NO_3_ | 90 | -50.00 | -41.67 |
| Bradford et al., 2001 | Temperate forest | UK | 50°55' N | 1˚21' W | 1 | 80 | 180 |  | 1190 | HNO_3_ | 11 | -67.10 | -65.42 |
| Bradford et al., 2001 | Temperate forest | UK | 50°55' N | 1˚21' W | 1 | 80 | 180 |  | 1190 | (NH_4_)_2_SO_4_ | 18.5 | -67.10 | -60.69 |
| Castro et al., 1993 | Temperate forest | USA | 29˚ N | 82°W | 4 | 26 |  | 21.7 | 1342 | Urea | 180 | -23.00 | -3.00 |
| Castro et al., 1992 | Temperate forest | USA | 43°30′N | 72°40'W | 3 |  | 762 |  |  | NH_4_Cl | 31.4 | -33.00 | -20.00 |
| Suwanwaree et al., 2005 | Temperate forest | USA | 42°24′N | 85°24′W | 1 |  | 288 | 9.7 | 890 | NH_4_NO_3_ | 100 | -31.60 | -12.70 |
| Steinkamp et al., 2001 | Temperate forest | Germany | 48°03'N | 8°22′E | 3 | 110 | 865 | 6 | 1200 | (NH_4_)_2_SO_4_ | 150 | -82.20 | -84.20 |
| Chan et al., 2005 | Temperate forest | USA | 41°36′N | 80°2′W | 8 | 80 | 390 |  | 1050 | NH_4_NO_3_ | 100 | -190.00 | -120.00 |
| Bowden et al., 2000 | Temperate forest | USA | 41°36′N | 80°2′W | 1 | 80 | 391 |  | 1050 | NH_4_NO_3_ | 100 | -154.38 | -127.53 |
| Vilarrasa-Nogué et al., 2019 | Temperate forest | Spanish | 40°32′N | 0°30′E | 14 | 14 |  | 15.1 | 333 | NH_4_NO_3_ | 50 | -115.00 | -13.00 |
| Chen et al., 2022 | Temperate forest | China | 33°36′N | 107°50′E | 7 |  | 1560 | 11.8 | 1075 | Urea | 30 | -245.00 | -264.80 |
| Chen et al., 2022 | Temperate forest | China | 33°36′N | 107°50′E | 7 |  | 1560 | 11.8 | 1075 | Urea | 150 | -245.00 | -232.00 |
| Yang et al., 2017 | Temperate forest | China | 31°54′N | 110°68′E | 5 |  | 133 | 11.6 | 638.3 | NaNO_3_ | 50 | -0.65 | -0.72 |
| Yang et al., 2017 | Temperate forest | China | 31°54′N | 110°68′E | 5 |  | 133 | 11.6 | 638.3 | NaNO_3_ | 150 | -0.65 | -0.45 |
| Yang et al., 2017 | Temperate forest | China | 31°54′N | 110°68′E | 5 |  | 133 | 11.6 | 638.3 | (NH_4_)_2_SO_4_ | 50 | -0.65 | -0.39 |
| Yang et al., 2017 | Temperate forest | China | 31°54′N | 110°68′E | 5 |  | 133 | 11.6 | 638.3 | (NH_4_)_2_SO_4_ | 150 | -0.65 | -0.33 |
| Yang et al., 2017 | Temperate forest | China | 31°54′N | 110°68′E | 5 |  | 133 | 11.6 | 638.3 | NH_4_NO_3_ | 50 | -0.65 | -0.47 |
| Yang et al., 2017 | Temperate forest | China | 31°54′N | 110°68′E | 5 |  | 133 | 11.6 | 638.3 | NH_4_NO_3_ | 150 | -0.65 | -0.38 |
| Yan et al., 2021 | Temperate forest | China | 42°24′N | 128°06′E | 2 |  | 738 | 3.6 | 720 | NH_4_NO_3_ | 50 | 0.00 | 0.00 |
| Håkansson et al., 2021 | Temperate forest | Norway | 57°00′N | 14°03′E | 8 | 25 |  | 7 | 750 | NH_4_NO_3_ | 150 | -37.00 | -32.00 |
| Håkansson et al., 2021 | Temperate forest | Norway | 57°00′N | 14°03′E | 8 | 25 |  | 7 | 750 | NH_4_NO_3_ | 300 | -37.00 | -28.00 |
| Håkansson et al., 2021 | Temperate forest | Norway | 57°00′N | 14°03′E | 8 | 25 |  | 7 | 750 | NH_4_NO_3_ | 450 | -37.00 | -17.00 |
| Chang et al., 2021 | Temperate forest | China | 29°34′N | 101°0′E | 4 |  | 3000 | 3.8 | 1940 | NH_4_NO_3_ | 8 | -256.00 | -364.10 |
| Chang et al., 2021 | Temperate forest | China | 29°34′N | 101°0′E | 4 |  | 3000 | 3.8 | 1940 | NH_4_NO_3_ | 40 | -256.00 | -194.10 |
| Chang et al., 2021 | Temperate forest | China | 29°34′N | 101°0′E | 5 |  | 3000 | 3.8 | 1940 | NH_4_NO_3_ | 8 | -349.20 | -470.40 |
| Chang et al., 2021 | Temperate forest | China | 29°34′N | 101°0′E | 5 |  | 3000 | 3.8 | 1940 | NH_4_NO_3_ | 40 | -349.20 | -266.80 |
| Chang et al., 2021 | Temperate forest | China | 29°34′N | 101°0′E | 6 |  | 3000 | 3.8 | 1940 | NH_4_NO_3_ | 8 | -309.20 | -405.70 |
| Chang et al., 2021 | Temperate forest | China | 29°34′N | 101°0′E | 6 |  | 3000 | 3.8 | 1940 | NH_4_NO_3_ | 40 | -309.20 | -241.10 |

Table S3 The reduction in CH_4_ uptake under N addition at site A

| Treatment | Decreases of CH_4_ uptake under N addition | |
| --- | --- | --- |
|  | N20 | N50 |
| SR | 0.17 | 0.31 |
| R | 0.13 | 0.11 |
| S | 0.04 | 0.20 |
| R/SR | 76.5% | 23.6% |

Table S4 Results (F value) of two-way ANOVAs on the effects of nitrogen additions (N), experimental year (Y), and their interactions (N × Y) effects on biotic and abiotic variables at site A and site B. ∗ denoted *P* < 0.05.

|  | Site A | | | Site B | | | |  |
| --- | --- | --- | --- | --- | --- | --- | --- | --- |
|  | N | Y | N × Y | | N | Y | N × Y | |
| Soil temperature | 0.67 | 1.65 | 1.21 | | 0.41 | 0.90 | 0.48 | |
| Soil moisture | 0.35 | 4.53^∗^ | 0.24 | | 0.21 | 3.67^∗^ | 0.88 | |
| NH_4_^+^-N (mg L^-1^) | 12.48^∗^ | 1.82 | 0.60 | | 21.69^∗^ | 0.74 | 1.04 | |
| NO_3_^-^-N (mg L^-1^) | 19.53^∗^ | 4.68^∗^ | 2.87 | | 33.75^∗^ | 1.59 | 2.36 | |
| pH | 1.95 | 1.66 | 0.69 | | 2.31^∗^ | 0.37 | 1.38 | |
| MBC (mg L^-1^) | 3.85^∗^ | 4.88^∗^ | 2.64 | | 1.25 | 0.93 | 0.86 | |
| MBN (mg L^-1^) | 1.77 | 4.51^∗^ | 1.39 | | 2.44 | 2.91 | 3.30 | |
| MBC:MBN | 2.21 | 0.86 | 1.55 | | 5.67^∗^ | 1.85 | 2.30 | |
| TOC (mg g^-1^) | 4.38^∗^ | 1.23 | 1.87 | | 6.70^∗^ | 2.52 | 1.49 | |
| DOC (mg kg^-1^) | 6.22^∗^ | 1.98 | 2.21 | | 5.13^∗^ | 2.33 | 1.87 | |
| DOC_root_(mg kg^-1^) | 9.37 | 14.14^∗^ | 2.80 | | / | / | / | |
| Soil respiration (umol m^-2^ s^-1^) | 8.50^∗^ | 5.98^∗^ | 3.56 | | / | / | / | |
| Rhizosphere respiration (umol m^-2^ s^-1^) | 10.51^∗^ | 5.11^∗^ | 1.39 | | / | / | / | |
| Heterotrophic respiration (umol m^-2^ s^-1^) | 4.71^∗^ | 3.59 | 2.02 | | / | / | / | |
| Fine root biomass (g m^-2^) | 9.12^∗^ | 36.21^∗^ | 5.83^∗^ | | / | / | / | |
| Coarse root biomass (g m^-2^) | 2.75 | 3.06 | 1.65 | | / | / | / | |
| Total root biomass (g m^-2^) | 3.34 | 31.72^∗^ | 2.18 | | / | / | / | |
| Root carbon concentration (%) | 5.13^∗^ | 26.31^∗^ | 2.25 | | / | / | / | |
| Root nitrogen concentration (%) | 2.33 | 13.47 | 1.24 | | / | / | / | |
| Litter mass (g m^-2^ yr^-1^) | 0.78 | 2.84 | 1.93 | | / | / | / | |

Reference list for reference source in Table S2 (there were 21 publications used in our meta-analysis).

1. Ambus, P. and Robertson, G.P., 2006. The Effect of Increased N Deposition on Nitrous oxide, Methane and Carbon dioxide Fluxes from Unmanaged Forest and Grassland Communities in Michigan. Biogeochemistry, 79(3): 315-337.
2. Aronson, E.L., Vann, D.R. and Helliker, B.R., 2012. Methane flux response to nitrogen amendment in an upland pine forest soil and riparian zone. Journal of Geophysical Research: Biogeosciences, 117(G3): G03012.
3. Bowden, R.D., Rullo, G., Stevens, G.R. and Steudler, P.A., 2000. Soil fluxes of carbon dioxide, nitrous oxide, and methane at a productive temperate deciduous forest. Journal of Environmental Quality, 29(1): 268-276.
4. Bradford, M.A., Wookey, P.A., Ineson, P. and Lappin-Scott, H.M., 2001. Controlling factors and effects of chronic nitrogen and sulphur deposition on methane oxidation in a temperate forest soil. Soil Biology & Biochemistry, 33(1): 93-102.
5. Castro, M.S., Peterjohn, W.T., Melillo, J.M., Steudler, P.A., Gholz, H.L. and Lewis, D., 1994. Effects of Nitrogen-Fertilization on the Fluxes of N2o, Ch4, and Co2 from Soils in a Florida Slash Pine Plantation. Canadian Journal of Forest Research-Revue Canadienne De Recherche Forestiere, 24(1): 9-13.
6. Castro, M.S., Steudler, P.A., Melillo, J.M., Aber, J.D. and Millham, S., 1992. Exchange of N2o and Ch4 between the Atmosphere and Soils in Spruce-Fir Forests in the Northeastern United-States. Biogeochemistry, 18(3): 119-135.
7. Chan, A.S.K., Steudler, P.A., Bowden, R.D., Gulledge, J. and Cavanaugh, C.M., 2005. Consequences of nitrogen fertilization on soil methane consumption in a productive temperate deciduous forest. Biology and Fertility of Soils, 41(3): 182-189.
8. Chang, R., Liu, X., Wang, T., Li, N. and Bing, H., 2021. Stimulated or Inhibited Response of Methane Flux to Nitrogen Addition Depends on Nitrogen Levels. Journal of Geophysical Research-Biogeosciences, 126(11).
9. Chen, Y., Yin, S., Shao, Y., Zhang, Q. and Zhang, K., 2022. Greenhouse gas fluxes from riparian forest soil depend on the responses of microbes to nitrogen and phosphorus additions. Applied Soil Ecology, 173.
10. Geng, J., Cheng, S., Fang, H., Yu, G., Li, X., Si, G., He, S. and Yu, G., 2017. Soil nitrate accumulation explains the nonlinear responses of soil CO_2_ and CH_4_ fluxes to nitrogen addition in a temperate needle-broadleaved mixed forest. Ecological Indicators, 79: 28-36.
11. Hakansson, C., Hedwall, P.-O., Stromgren, M., Axelsson, M. and Bergh, J., 2021. Effects of fertilization on soil CH4 and N2O fluxes in young Norway spruce stands. Forest Ecology and Management, 499.
12. Jassal, R.S., Black, T.A., Roy, R. and Ethier, G., 2011. Effect of nitrogen fertilization on soil CH_4_ and N_2_O fluxes, and soil and bole respiration. Geoderma, 162(1-2): 182-186.
13. Kim, Y.S., Imori, M., Watanabe, M., Hatano, R., Yi, M.J. and Koike, T., 2012. Simulated nitrogen inputs influence methane and nitrous oxide fluxes from a young larch plantation in northern Japan. Atmospheric Environment, 46: 36-44.
14. Sitaula, B.K., Bakken, L.R. and Abrahamsen, G., 1995. Ch4 Uptake by Temperate Forest Soil - Effect of N Input and Soil Acidification. Soil Biology & Biochemistry, 27(7): 871-880.
15. Steinkamp, R., Butterbach-Bahl, K. and Papen, H., 2001. Methane oxidation by soils of an N limited and N fertilized spruce forest in the Black Forest, Germany. Soil Biology & Biochemistry, 33(2): 145-153.
16. Steudler, P.A., Bowden, R.D., Melillo, J.M. and Aber, J.D., 1989. Influence of Nitrogen-Fertilization on Methane Uptake in Temperate Forest Soils. Nature, 341(6240): 314-316.
17. Suwanwaree, P. and Robertson, G.P., 2005. Methane Oxidation in Forest, Successional, and No-till Agricultural Ecosystems. Soil Science Society of America Journal, 69(6): 1722-1729.
18. Vilarrasa-Nogue, M., Teira-Esmatges, M.R., Villar, J.M. and Rufat, J., 2019. Effect of N dose on soil GHG emissions from a drip-fertigated olive (Olea europaea L.) orchard. Sci Total Environ, 677: 350-361.
19. Wang, Y., Cheng, S., Fang, H., Yu, G., Yang, X., Xu, M., Dang, X., Li, L. and Wang, L., 2016. Relationships between ammonia-oxidizing communities, soil methane uptake and nitrous oxide fluxes in a subtropical plantation soil with nitrogen enrichment. European Journal of Soil Biology, 73: 84-92.
20. Yan, G., Xing, Y., Liu, G., Huang, B. and Wang, Q., 2021. Precipitation Pattern Regulates Soil Carbon Flux Responses to Nitrogen Addition in a Temperate Forest. Ecosystems, 24(7): 1608-1623.
21. Yang, X., Wang, C. and Xu, K., 2017. Response of soil CH_4_ fluxes to stimulated nitrogen deposition in a temperate deciduous forest in northern China: A 5-year nitrogen addition experiment. European Journal of Soil Biology, 82: 43-49.
